# Suppression of ITPKB degradation by Trim25 confers TMZ resistance in glioblastoma through ROS homeostasis

**DOI:** 10.1038/s41392-024-01763-x

**Published:** 2024-03-04

**Authors:** Yuanliang Yan, Shangjun Zhou, Xi Chen, Qiaoli Yi, Songshan Feng, Zijin Zhao, Yuanhong Liu, Qiuju Liang, Zhijie Xu, Zhi Li, Lunquan Sun

**Affiliations:** 1 Department of Pharmacy, Xiangya Hospital, Central South University, Changsha, 410008 China; 2 Department of Neurosurgery, Xiangya Hospital, Central South University, Changsha, 410008 China; 3 Department of Pathology, Xiangya Hospital, Central South University, Changsha, 410008 China; 4 Xiangya Cancer Center, Xiangya Hospital, Central South University, Changsha, 410008 China; 5 Key Laboratory of Molecular Radiation Oncology Hunan Province, Changsha, 410008 China; 6 Institute of Cancer Research, National Clinical Research Center for Geriatric Disorders (Xiangya), Xiangya Hospital, Central South University, Changsha, 410008 China

**Keywords:** Cell biology, Biomarkers

## Abstract

Temozolomide (TMZ) represents a standard-of-care chemotherapeutic agent in glioblastoma (GBM). However, the development of drug resistance constitutes a significant hurdle in the treatment of malignant glioma. Although specific innovative approaches, such as immunotherapy, have shown favorable clinical outcomes, the inherent invasiveness of most gliomas continues to make them challenging to treat. Consequently, there is an urgent need to identify effective therapeutic targets for gliomas to overcome chemoresistance and facilitate drug development. This investigation used mass spectrometry to examine the proteomic profiles of six pairs of GBM patients who underwent standard-of-care treatment and surgery for both primary and recurrent tumors. A total of 648 proteins exhibiting significant differential expression were identified. Gene Set Enrichment Analysis (GSEA) unveiled notable alterations in pathways related to METABOLISM_OF_LIPIDS and BIOLOGICAL_OXIDATIONS between the primary and recurrent groups. Validation through glioma tissue arrays and the Xiangya cohort confirmed substantial upregulation of inositol 1,4,5-triphosphate (IP3) kinase B (ITPKB) in the recurrence group, correlating with poor survival in glioma patients. In TMZ-resistant cells, the depletion of ITPKB led to an increase in reactive oxygen species (ROS) related to NADPH oxidase (NOX) activity and restored cell sensitivity to TMZ. Mechanistically, the decreased phosphorylation of the E3 ligase Trim25 at the S100 position in recurrent GBM samples accounted for the weakened ITPKB ubiquitination. This, in turn, elevated ITPKB stability and impaired ROS production. Furthermore, ITPKB depletion or the ITPKB inhibitor GNF362 effectively overcome TMZ chemoresistance in a glioma xenograft mouse model. These findings reveal a novel mechanism underlying TMZ resistance and propose ITPKB as a promising therapeutic target for TMZ-resistant GBM.

## Introduction

Glioma, the most prevalent primary malignant tumor of the central nervous system, is associated with a poor prognosis and a low median survival time.^1^ Glioblastoma (GBM), the most aggressive subtype of glioma, accounts for approximately 50% of cases and poses a formidable challenge for the clinical and scientific communities.^2^ The current standard of care for GBM involves radiotherapy in combination with the DNA alkylating agent temozolomide (TMZ).^3^ In 2005, a randomized controlled trial reported improved survival rates for patients with GBM who received TMZ in conjunction with radiotherapy, with patient survival increasing from 12.1 to 14.6 months.^4^ Despite progress in both basic and clinical research, the blood-brain barrier limits the effectiveness of GBM therapies, resulting in uniformly poor median survival rates in the past few decades.^5^ After initial treatment, most patients with GBM (nearly 90%) experience recurrence in situ.^6^ Unfortunately, there have been no efficient treatment options for recurrent GBM. Therefore, there is an urgent need to further understand the molecular pathology of GBM, identify novel drug targets, and develop new treatments for glioma.

Reactive oxygen species (ROS) play a double role in human cancers, exerting both tumor-promoting and tumor-suppressing effects.^7^ ROS can modulate malignant behaviors by inducing oncogenic perturbations, altering mitochondrial activity, increasing oxidative stress, and improving ROS-dependent signaling.^8^ In general, chemotherapy agents exert their anticancer effects by altering redox homeostasis.^9^ In addition to DNA alkylation, TMZ-induced cytotoxicity in glioma cells is mediated by increased ROS levels or inhibition of antioxidant systems.^10^ Studies have shown that TMZ treatment causes an alteration of ROS homeostasis, predominantly with the accumulation of ROS.^11^ Chemoresistance to TMZ is linked to oxidative stress insensitivity and hydrogen peroxide challenges, but treatment with the oxidant increased TMZ-dependent ROS generation and reversed chemoresistance.^12^ Polyphyllin VII extracted from *Paris polyphylla* var exhibited a cytotoxic effect by inhibiting ROS-induced AKT/mTORC1 activity and sensitized glioma cells to TMZ.^13^ Sirtuin 1 inhibition sensitizes glioma cells to TMZ treatment by facilitating intracellular ROS generation.^14^ To maintain glioma stem cells (GSCs), low levels of ROS mediated by prohibitin protect GSCs from ionizing radiation (IR)-induced cell death and promote GBM therapeutic resistance.^15^ These observations indicate that the low level of ROS contributes significantly to GBM treatment resistance; however, the underlying mechanisms remain to be explored.

Inositol-1,4,5-triphosphate (IP3) kinase B (ITPKB) is a ubiquitously expressed lipid kinase that phosphorylates IP3, an intracellular secondary messenger that stimulates calcium release and mitochondrial ATP production.^16^ GNF362, a pharmacological inhibitor of ITPKB with high potency and selectivity, increases intracellular Ca^2+^ and apoptosis of activated T cells.^4,17^ Emerging evidence supports that dysregulated ITPKB is associated with resistance to chemotherapy in cancers through the activation of NADPH oxidase (NOX). For example, ITPKB provides a metabolic advantage to cancer cells, leading to cisplatin-resistant cancer cell survival by controlling redox homeostasis.^18,19^ The ITPKB product IP4 can limit NOX4 activity by competitive binding with the NADPH cofactor, thus controlling cisplatin-induced ROS and conferring resistance to cisplatin in human cancer.^20^ Furthermore, the phosphorylation of ITPKB in serine 174 by CAMK2G, regulated by ROS, is a critical vulnerability that drives cisplatin resistance by facilitating adaptive redox homeostasis after cisplatin treatment.^21^ In this, we identified ITPKB as a novel target for resistance to TMZ by proteomic analyses of primary and recurrent tissue from GBM patients. Upregulated expression of ITPKB in GBM cells is positively correlated with TMZ resistance and poor survival both in vivo and in vitro. The ubiquitination of ITPKB in K793 and K818 is essential for its proteasome degradation through the E3 ubiquitin ligase enzyme Trim25. Genetic and pharmacological inhibition of ITPKB significantly inhibits tumor growth. It improves the TMZ chemotherapy response by ROS upregulation, indicating that Trim25-mediated ubiquitination of ITPKB may be an important regulatory mechanism in the pathogenesis and treatment of GBM.

## Materials and methods

### Cell culture, plasmids, and antibodies

HEK293T, T98G, U118 and U87 cells were obtained from the Cancer Research Institute, Central South University, China, and identified by short tandem repeat genotyping as in our previous study. TMZ-resistant cell lines, T98G-R, U118-R, and U87-R, were established by continuous stepwise selection using increasing concentrations of TMZ, as previously reported.^22,23^ A pair of radiation-sensitive and resistant GBM cells (U251 and U251-IR cells) were used to investigate the role of the target gene in radioresistance of GBM.^24^ Cells were cultured in Dulbecco’s modified eagle medium (DMEM) with 10% fetal bovine serum (FBS) and 1% penicillin/streptomycin.

ITPKB knockdown T98G-R, U118-R and U87-R cells were generated using lentivirus derived from a lentiviral packaging plasmid, psPAX2, and the plasmid pMD2.G containing VSV-G. The ITPKB shRNA sequences (#1: 5′-ATAACATCCTGATCGCCTATC-3′, targeting CDS; #2: 5′-ACCAGAAAGTGGGCATGTTTG-3′, targeting CDS) were obtained from Sigma (USA). Trim25 siRNA sequences (#1: 5′-GGGTCAACAGCAAGTTTGA-3′; #2: 5′-CAGCAAGCTTCCCACGTTT-3′) were purchased from Ribobio (China). All flag-tagged ITPKB truncated mutants (WT, 1-768, 1-800, 768-946, and 800-946) and V5-tagged Trim25 were purchased from Sangon Biotech (China). ITPKB shRNA-resistant Flag-ITPKB WT (3×Flag tag at the N-terminus), Flag-ITPKB DN (kinase-dead D897N), and Trim25 S100D and S100A plasmids were obtained from TsingkeBiotechnology (China). Flag-ITPKB truncations with K793R and/or K818R mutations were acquired from Sangon Biotech (China). All point mutations and truncations were performed using a site-directed mutagenesis method based on PCR and confirmed by Sanger sequencing.

Anti-ITPKB (12816-1-AP) and anti-Trim25 (67314-1-1g, 12573-1-AP) antibodies were procured from Proteintech. Anti-Flag (D6W5B), anti-V5 (E9H8O), anti-ubiquitin (P4D1), and rabbit mAb IgG antibody (DA1E) were obtained from Cell Signaling. Anti-HA (H3663) was obtained from Sigma. AP2A1 and CLIP1 siRNAs were purchased from Ribobio (China). AP2A1 (JE40-38) and CLIP1 antibodies (JE63-82) were acquired from Huabio. Anti-cleaved caspase-3 antibody (ab2302) was obtained from Abcam. The anti-cleaved caspase-9 antibody (#9505) was obtained from Cell Signaling. Ki-67 (ZM-0166) antibody was procured from ZSGB-BIO. Anti-actin (sc-8432) was obtained from Santa Cruz. Pierce protein A/G magnetic beads (88802) were purchased from ThermoFisher. Anti-FLAG® M2 magnetic beads (M8823) were sourced from Sigma. Puromycin (S7417) was obtained from Selleck. The following inhibitors were used: MG132 (Selleckchem: S2619), Cycloheximide (Sigma: 01810), GNF362 (HY-126750, MedChemExpress), and the NOX1/4 inhibitor GKT137831 (GKT831, MedChemExpress).

### Transfection

For viral infections, Lipofectamine™ 3000 transfection reagent (ThermoFisher, L3000001) was used to cotransfect the shRNA vector, the envelope plasmid pMD2.G, and the packaging plasmid psPAX2 in HEK293T cells. The lentivirus media was collected and used to infect cells in the presence of polybrene (MedChemExpress, HY-112735, 8 μg/mL) after 48 h of transfection. Subsequently, cells were harvested after 48 h and selected in puromycin to generate a pure cell population.

### GBM patients and mass spectrometry

The Ethical Review Committee of the Xiangya Hospital approved the study protocol and the acquisition of tissue specimens. Patients with histologically confirmed GBM, who underwent a similar first-line treatment scheme that involved maximal surgical resection followed by radiotherapy with concomitant and adjuvant TMZ-based chemotherapy, and subsequently underwent surgery for both primary and recurrent diseases at the Xiangya Hospital (Hunan, China) were recruited. Fresh GBM tissue samples from all primary and recurrent surgery patients were promptly stored in liquid nitrogen. Ultimately, six pairs of GBM tissue specimens were collected by surgical resection. All patients provided written informed consent to the research use of tissue specimens. The TMT-labeling HPLC–MS/MS proteome analysis was conducted by Jingjie PTM BioLabs (Hangzhou, China) to identify and quantify the peptides and their abundance. Significantly differentially expressed proteins were selected based on the following criteria: *P* < 0.05 and an absolute fold change >1.2. The GSEA pathway enrichment analysis (c2.cp.v7.2.symbols.gmt [Curated]) was performed for all significantly differentially expressed proteins.

### Immunohistochemical staining

For the immunohistochemical staining experiment, 19 pairs of GBM specimens were obtained from patients who received TMZ and underwent primary and recurrent surgical treatment at Xiangya Hospital. Human glioma tissue arrays (HBraG180Su02) were purchased from Shanghai Outdo Biotech Co. Ltd. The tissue sections were dehydrated, repaired with citrate antigen, and blocked with 5% goat serum for 15 min at room temperature. The tissue sections were stained with a diluted primary anti-ITPKB antibody at 4 °C overnight. Subsequently, the tissue sections were incubated with DAB, counterstained with hematoxylin, and dehydrated with ethanol. The images were captured and processed using ImageScope software (Leica Microsystems). Two independent pathologists examined and scored all sections in a double-blind manner. Quantitative scoring of the proportion of positive cells was performed as follows: 1, less than 25%; 2, 25–50%; 3, 50–75%; and 4, more than 75%. The stain intensity was ranked on a scale of 0–3 (0, negative; 1, light brown; 2, medium brown; and 3, dark brown). The histological score for each section was calculated using the formula (Total score = proportion score × intensity score) and divided into high or low based on the median total score of 4.

### Immunoprecipitation and western blot assays

Cells were lysed in a NETN buffer (20 mM Tris-HCl, pH 8.0, 500 mM NaCl, 1 mM EDTA, 0.5% Nonidet P-40 with protease inhibitors) that was supplemented with protease inhibitors as previously reported.^25^ After centrifugation at 14,000 × *g* for approximately 15 min, the supernatant was incubated with Anti-FLAG® M2 magnetic beads or specified antibodies along with protein A/G magnetic beads, rotating overnight at 4 °C. The beads were washed three times with NETN buffer, and the samples were eluted with 4× SDS loading buffer before immunoblotting with the indicated antibodies. For SDS-PAGE immunoblotting, PVDF membranes (Millipore) were blocked in 5% skim milk in TBST buffer and then incubated with primary antibodies overnight at 4 °C, as previously described. The protein bands were quantified using Image Lab software version 5.0 (Bio-Rad).

### Ubiquitination assays

For denaturing immunoprecipitation for ubiquitination, cells were lysed using 100 µL of NETN lysis buffer (62.5 mM Tris-HCl, pH 6.8, 2% SDS, 10% glycerol, 20 mM NEM, and 1 mM iodoacetamide). After boiling for 15 min, the reactions were diluted 10 times with the NETN buffer containing protease inhibitors, 20 mM NEM, and 1 mM iodoacetamide and then centrifuged to remove cell debris. Subsequently, the cell extracts were subjected to immunoprecipitation and analyzed by blotting with the indicated antibodies.

For in vitro ubiquitination assays, 2 μg V5-Trim25 or Flag-ITPKB purified from HEK293T cells was used with ultrasonication. The E3 ligase V5-Trim25 was mixed with 10 U of FastAP™ Thermosensitive alkaline phosphatase (Thermo Scientific, #EF0654). The E3 ligase complex was then mixed with 2 μg Flag-ITPKB purified from HEK293T cells and reacted with 15 μg wild-type ubiquitin (Boston Biochem, U-100H-10M), 550 ng of E1 (Uba1, Boston Biochem, E-305-025), and 850 ng of E2 (UbcH5b, Boston Biochem, E2-622-100).^26^ The complex was incubated in a reaction buffer (50 mM Tris-HCl, pH 7.4, 5 mM MgCl2, 1 mM dithiothreitol [DTT], and 2 mM ATP) at 37°C for 1 h. The reaction products were analyzed by SDS-PAGE and immunoblotting.

### In situ PLA assay

Following the manufacturer’s instructions, the Duolink in situ PLA kit (Sigma, # DUO92101) was used to investigate protein-protein interactions in cells. TMZ-sensitive and resistant cells were collected and seeded directly onto the cover glass. After washing with PBS, the samples were fixed in 3% paraformaldehyde for 15 min and permeabilized in a 0.5% Triton-X solution for 5 min at room temperature. Subsequently, the samples were incubated with Trim25 and ITPKB antibodies for 1 h after blocking. Duolink PLA Probe and Duolink Ligation buffer were added over 1 h or 30 min at 37 °C, respectively. The samples were incubated with Amplification buffer for 100 min at 37 °C and stained with DAPI to visualize nuclear DNA. The coverslips were mounted onto glass slides with an antifade solution. The ZEISS LSM 9 family of confocal microscopes was used to visualize the PLA signal dots, which were counted using ImageJ.

### Quantitative real-time PCR

mRNA was extracted using TRIzol reagent (Invitrogen). The RNA samples were reverse-transcribed using SuperScript™ II reverse transcriptase (Invitrogen) and amplified with Power SYBR Green PCR Master Mix (Takara). The primers used for the SYBR Green assays were as follows: ITPKB-F:5′-TCTCCTCATCCTACGAAGACTCA-3′; ITPKB-R: 5′-GCTCACTCTAGGTTTCTGCTGG-3′; GAPDH-F: 5′-CAGCCTCAAGATCATCAGCA-3′; and GAPDH-R: 5′-TGTGGTCATGAGTCCTTCCA-3′. Real-time amplification was performed on an ABI Prism 7000 SDS (Applied Biosystems). The quantification of gene expression was calculated based on the 2^−ΔΔCT^ value normalized to GAPDH.

### Colony formation and CCK8 assays

To assess cell survival in TMZ or GNF362, 1 × 10^3^ T98G-R, U118-R or U87-R cells that stably express the indicated constructs were seeded in triplicate in each well of 6-well plates for 24 h before being treated with the indicated dose of drugs for 10-15 days. The colonies were fixed with 100% ethanol, stained with a 0.006% crystal violet solution, and subsequently counted. The results were normalized to the plating efficiencies. The IC_50_ of glioma cells was determined using the Cell Counting Kit‐8 following the manufacturer’s protocols (MedChemExpress). Cells were seeded at a density of 5 × 10^3^/well and treated with various concentrations of TMZ. After treatment for 4 days, 10 μL of CCK‐8 reagent was added to each well, and the absorbance was analyzed at 450 nm using a microplate reader (PerkinElmer), with wells without cells as blanks. The IC_50_ of the cells was determined using dose-response curves generated by GraphPad Prism software.

### ROS measurement

ROS analysis was conducted using 2′,7′-Dichlorofluorescein diacetate (DCFH-DA) (35845, Sigma). Cells from each group were digested with EDTA-free trypsin, washed three times, and then treated with DCFH-DA at a final concentration of 10 µM. Subsequently, cells were incubated for 30 min and gently mixed by inverting every 3–5 min. The fluorescence intensity was measured using a CytoFLEX Research Flow Cytometry instrument (Beckman Coulter) at 488 nm excitation and 525 nm emission wavelengths. Data were analyzed using FlowJo 10 software.

### NOX activity assay

The NOX activity assay was conducted using the NADH oxidase activity assay kit (D799203, Sangon) following the manufacturer’s instructions. Briefly, cells were homogenized, and the supernatant obtained after centrifugation was used to determine NOX activity and protein concentration (Cpr, mg/mL). Reaction reagents were gradually mixed in a 1 mL glass colorimetric plate, and the initial absorbance A1 was recorded at a wavelength of 600 nm for 20 s. NOX activity involves the reduction of a colored substrate to a colorless product. Subsequently, the colorimetric dish and reaction solution were quickly placed in a 37 °C water bath, and the absorbance A2 was accurately recorded at 1 min and 20 s. NOX activity was calculated by normalizing NOX (U/mg protein) based on protein concentration.

### IP4 assay

The detection of IP4 was conducted using the IP4 ELISA Kit (ml060364, Mlbio). The microplate wells were coated with an HRP-labeled IP4 antibody. Subsequently, samples were added to each well and incubated for 10 min at 37°C. After incubation, the coating solution was aspirated, and the plate was washed three times with a wash buffer to remove any unbound antibodies. After thorough washing, a substrate solution of TMB was added to induce color development. The TMB, catalyzed by the HRP enzyme, transitioned from blue to yellow under the influence of an acid. The absorbance (OD value) was measured at a wavelength of 450 nm using a microplate reader. The concentration of human IP4 in the samples was determined by calculation using a standard curve.

### Apoptosis assay

Cell apoptosis was evaluated using the Annexin V-FITC apoptosis kit (C1062M, Beyotime) according to the manufacturer’s instructions. Cells were collected and resuspended in a 195 μL Annexin V-FITC binding buffer, followed by incubation with 5 μL Annexin V-FITC and 10 μL propidium iodide for 10 min at room temperature in the dark. Subsequently, cells from each sample were analyzed using CytoFLEX Research Flow Cytometry (Beckman Coulter). Data were processed using FlowJo 10 software, and the upper right quadrants (Annexin V+/PI+) indicate late apoptotic cells.

### Tumor xenograft

The experiments were conducted with the approval of the Animal Care and Use Committee of Central South University (China). Control or ITPKB knockdown T98G-R cells (1 × 10^7^) were subcutaneously injected into the flanks of Athymic BALB/c nude mice (age 4 weeks) with 30% growth factor-reduced Matrigel (BD Biosciences). Mice-bearing tumors of approximately 50 mm^3^ were randomly divided into two groups: the control group (saline) and the TMZ group (20 mg/kg, every 3 days) by intraperitoneal injection. To assess the combined treatment of GNF362 and TMZ, the mouse tumor model was established by subcutaneous injection of 1 × 10^7^ T98G-R cells. Mice in the GNF362 group were treated with 200 µg GNF362 (every 3 days) by gavage. Tumor volume was measured every 3 days using calipers from the initiation of treatment and calculated using the formula length × width^2^. Mice were sacrificed for tumor dissection on day 35 after starting treatment.

### Quantification of H_2_O_2_

H_2_O_2_ levels were assessed using an H_2_O_2_ assay kit (Beyotime). Mouse tumor tissue (20 mg) was added to a 200 μL lysis buffer, homogenized, and centrifuged to obtain the supernatant. The supernatant (50 μL) and 100 μL of the test solution were combined in a tube at room temperature for 30 min, and the absorbance values at 560 nm were measured using a microplate reader (PerkinElmer). The absorbance values were calibrated to a standard concentration curve to calculate the concentration of H_2_O_2_.

### Statistical analysis

Data are presented as mean ± SD (*n* ≥ 3) and were analyzed using GraphPad Prism 6 software (GraphPad Software). The significance of the differences between the groups with continuous data was assessed using the Student’s t-test. Spearman’s analysis was used to examine the relationship between ITPKB expression and TMZ IC_50_. Overall survival (OS) and progression-free survival (PFS) were calculated using Kaplan–Meier analysis and compared with the log-rank test. *p*-values less than 0.05 were considered statistically significant.

## Results

### Association of ITPKB with glioma malignancy

**Fig. 1:**
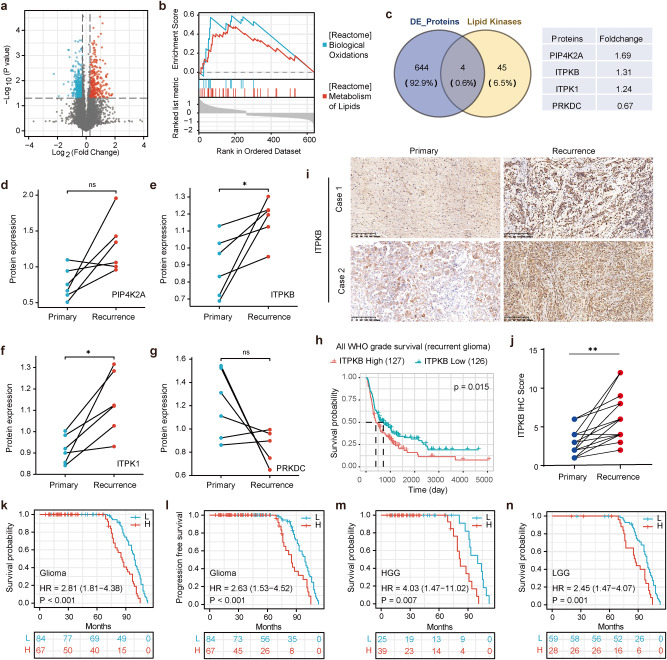
Differential expression analysis of the protein ITPKB in primary and recurrent glioma patients. **a** Six pairs of GBM tissues from glioma patients who received standard TMZ treatment and underwent primary and recurrent surgical treatment, were analyzed by mass spectrometry. A volcano plot represented the differential expression of proteins with a condition of *P* < 0.05 and an absolute fold change > 1.2. **b** GSEA pathway enrichment analysis was performed based on the identified differentially expressed proteins between the primary and recurrent groups in the tumors. **c** Lipid kinases and differentially expressed proteins depicted in a Venn diagram. **d**–**g** Expression profiles of differentially expressed lipid kinases (PIP4K2A, ITPKB, ITPK1, and PRKDC) in the proteomic analysis of primary and recurrent GBM tissue from six patients. **h** The prognostic significance of ITPKB in recurrent glioma patients was analyzed using the CGGA database (http://www.cgga.org.cn/). **i**, **j** IHC staining of ITPKB was performed in 19 pairs of primary/recurrent GBM patient specimens who received TMZ chemotherapy at Xiangya Hospital. The represented images are shown and quantification was analyzed by t-test. **k**, **l** IHC staining of ITPKB was analyzed in human glioma tissue arrays to assess the ITKPB survival values. The high or low group was reduced by the median total score of 4. The association of ITPKB with progression-free survival and overall survival of glioma patients was assessed using the log-rank test. **m**, **n** Survival subgroup analysis was performed using the log-rank test in HGG and LGG patients. Statistical significance is shown as: *p < 0.01, **p < 0.001, ns nonsignificant

TMZ, in conjunction with radiotherapy, followed by surgical resection, has become the standard therapy for glioma globally, where drug resistance poses a significant challenge in GBM. To reveal potential biomarkers, novel drug targets, and the underlying mechanisms of TMZ resistance, six pairs of GBM patients who underwent standard-of-care treatment and surgery for both primary and recurrent diseases were enrolled. The tandem mass tag (TMT)-based proteomic analysis identified 7,524 proteins, with 266 proteins significantly upregulated and 382 downregulated between the recurrence and primary groups (*p* < 0.05 and fold change > 1.2) (Fig. 1a and Supplementary Table 1). Functional enrichment analysis of differentially expressed proteins revealed that BIOLOGICAL_OXIDATIONS and METABOLISM_OF_LIPIDS pathways were overrepresented in proteins upregulated in recurrent tumors (Fig. 1b and Supplementary Fig. 1a).

During the past four decades, the lipid peroxidation process has been shown to play crucial roles in cell biology and human health, wherein lipid kinases catalyze the production of lipids.^27^ By comparing the proteomic data of our study and the lipid kinases of KiNativ,^28^ a Venn diagram reveals four intersected kinases (PIP4K2A, ITPKB, ITPK1, and PRKDC) (Fig. 1c), which may be associated with glioma recurrence. Further validation showed that only ITPKB and ITPK1 were consistently highly expressed in recurrent samples compared to primary ones (Fig. 1d–g). Considering the oncological relevance, ITPKB was selected for further investigation. ITPKB, an essential cellular enzyme, catalyzes the inositol phosphate phosphorylation of the second messenger inositol 1,4,5-trisphosphate (IP3) to inositol 1,3,4,5-tetrakisphosphate (IP4), regulating intracellular calcium fluxes.^17^ Data from the Chinese Glioma Genome Atlas database (CGGA, http://www.cgga.org.cn/)^29^ demonstrated that high ITPKB expression was correlated with a poorer survival probability (Fig. 1h). Western blotting of the six pairs of GBM patients validated that ITPKB was highly expressed in recurrent samples compared to primary samples (Supplementary Fig. 1b). Pancancer analysis using the UALCAN platform^30^ revealed that ITPKB was expressed at significantly higher levels in pancreatic and GBM tumors compared to normal tissues (Supplementary Fig. 1c). To validate these findings, an additional 19 pairs of glioma patient tissues from Xiangya Hospital were analyzed, confirming the higher expression of ITPKB in the recurrent group compared to the primary group (Fig. 1i, j). Furthermore, tissue arrays collected from 151 glioma cases revealed that the group with higher expression of ITPKB had poorer overall survival (median OS: 86 vs. 99 months, *p* < 0.001) and PFS (median PFS: 85 vs. 99 months, *p* < 0.001) than the lower expression group (Fig. 1k, l). The stratified analysis showed that higher ITPKB expression predicted poorer OS in patients with high-grade glioma (HGG) and low-grade glioma (LGG) (Fig. 1m, n). These findings suggest a strong association of ITPKB with GBM recurrence and patient survival.

### Upregulation of ITPKB in TMZ-resistant GBM

In our study, patients diagnosed with pathologically confirmed GBM underwent standard first-line treatment, which included maximal surgical resection followed by TMZ-based chemotherapy with concurrent radiotherapy. Clinically, treatment resistance is identified as a primary factor contributing to glioma recurrence. To explore the role of ITPKB in the radioresistance of GBM, a pair of radiation-sensitive and resistant GBM cells (U251 and U251-IR) were used. The expression of the ITPKB protein did not differ significantly between radiosensitive and radioresistant GBM cells (Supplementary Fig. 2a). Therefore, we propose a direct association between elevated ITPKB expression in relapsed patients and TMZ resistance.

**Fig. 2:**
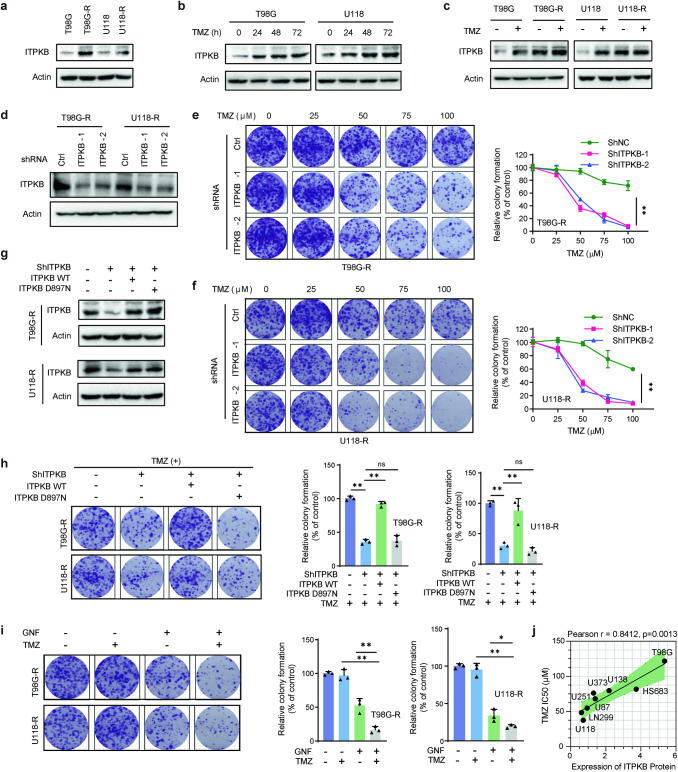
ITPKB-mediates TMZ sensitivity in GBM cells dependent on its kinase activity. **a** The expression of ITPKB was assessed in TMZ-sensitive (T98G, U118) and resistant (T98G-R, U118-R) cells using Western Blot. **b** T98G and U118 cells were treated with TMZ for indicated durations, and the cell lysates were blotted with specified antibodies. **c** After 48 h of TMZ treatment, lysates were collected from both TMZ-sensitive (T98G, U118) and resistant (T98G-R, U118-R) cells. Subsequently, these cell lysates were blotted with the indicated antibodies. **d** Construction of ITPKB-depleted TMZ-resistant glioma cell lines involved the transduction of T98G-R and U118-R cells with lentivirus encoding control (Ctrl) or ITPKB shRNAs. Cell lysates were then blotted with the specified antibodies. **e**, **f** Glioma cells from **d** were subjected to treatment with indicated doses of TMZ (0, 25, 50, 75, 100 μM) for 10-15 days. Cell survival was determined by colony formation assay. Error bars represent ± SD from three independent experiments. **g** TMZ-resistant glioma cells stably expressing Ctrl or ITPKB shRNA-1 were transiently transfected with the wild-type (WT) and kinase-dead mutant D897N of ITPKB. Subsequently, the cells were lysed, and Western blot analysis was performed using the specified antibodies. **h** Glioma cells from **g** were treated with 100 μM TMZ for 10–15 days, and cell survival was determined by colony formation assay. Error bars represent ± SD from three independent experiments. **i** TMZ-resistant glioma cells were treated with 20 μM GNF362 and/or 25 μM TMZ, and cell survival was determined by colony formation assay. Error bars represent ± SD from three independent experiments. **j** The expression of ITPKB in glioma cell lines (U251, U87, U118, U373, U138, LN299, HS683, and T98G) was assessed by western blot, and IC_50_ of each cell line was calculated after the addition of gradient concentration of TMZ for 96 h. The correlation between ITPKB expression and IC_50_ was analyzed by Pearson’s test in a panel of glioma cell lines. Statistical significance is indicated as: *p < 0.01, **p < 0.001, ns nonsignificant

Using two TMZ-resistant cell lines (U118-R, T98G-R) (Supplementary Fig. 2b, c), we observed a higher expression of the ITPKB protein in TMZ-resistant cells compared to sensitive glioma cells (U118, T98G) (Fig. 2a). In TMZ-sensitive T98G and U118 cells, TMZ treatment induced significant expression of ITPKB in a time-dependent manner (Fig. 2b). In contrast, this induction was not observed in TMZ-resistant T98G-R and U118-R cells (Fig. 2c, Supplementary Fig. 2d), suggesting that TMZ resistance may involve an adaptive process. Using two different ITPKB- shRNAs, we established T98G-R and U118-R cell lines with stable ITPKB knockdown (Fig. 2d). The colony formation assay revealed that ITPKB depletion significantly increased drug sensitivity in TMZ-resistant cell lines (Fig. 2e, f). Reexpression of ITPKB-WT, but not of the kinase-dead ITPKB-DN mutant, led to the reversion of survival inhibition in ITPKB-depleted cells (Fig. 2g, h).

GNF362 is a selective, potent, and orally bioavailable inhibitor of ITPKB.^17^ In TMZ-resistant cell lines, the combined use of GNF362 with TMZ significantly inhibited cell survival compared to the GNF362 or TMZ used alone (Fig. 2i). Furthermore, a positive correlation was validated between ITPKB protein expression and TMZ IC_50_ using a panel of eight glioma cell lines (Pearson *r* = 0.8412, *p* = 0.0013) (Fig. 2j). Considering the high heterogeneity of human glioblastoma, we further validated our findings in another TMZ-resistant U87-R GBM cell line, established in our previous study^23^ (Supplementary Fig. 2e). We showed that downregulation of ITPKB increased sensitivity to TMZ in U87-R cells (Supplementary Fig. 2f). Wild-type ITPKB could rescue enhanced sensitivity, but not the kinase-dead ITPKB-DN mutant control (Supplementary Fig. 2g). The combined use of GNF362 and TMZ significantly inhibited U87-R cell survival compared to GNF362 or TMZ used alone (Supplementary Fig. 2h). Collectively, these observations underscore the crucial role of ITPKB in TMZ resistance through its kinase activity.

### ITPKB-mediated TMZ resistance through regulation of ROS homeostasis

To unravel the underlying mechanism of ITPKB-mediated TMZ resistance, we used the Linkedomics database (http://www.linkedomics.org/login.php)^31^ to analyze the TCGA glioma dataset based on ITPKB expression. Analysis revealed a positive correlation between ITPKB expression and peroxisomal lipid metabolism and ROS-related signaling pathways, including Oxidative Stress Response, Biological Oxidations, and RHO GTPases Activate NADPH Oxidases (Supplementary Fig. 3a). Our proteomic analyses of six pairs of GBM patients also identified the enrichment of the BIOLOGICAL_OXIDATIONS pathway in recurrent tumors (Fig. 1b), further suggesting the involvement of ITPKB in regulating ROS homeostasis.

**Fig. 3:**
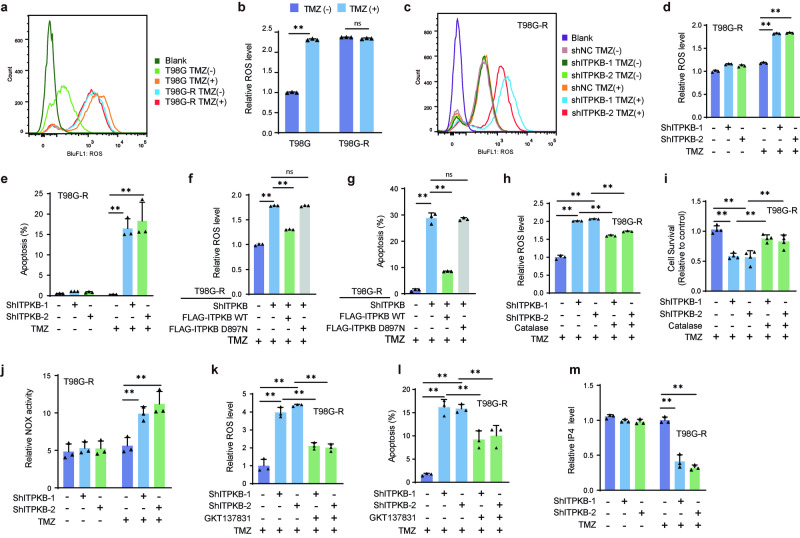
ITPKB participates in TMZ sensitivity through ROS homeostasis. **a**, **b** TMZ-sensitive and resistant cells were exposed to 100 μM TMZ for 48 h, and ROS levels were assessed using the DCFDA assay. Data, derived from three independent samples, are presented as mean fold change to control ± SD. **c**–**e** T98G-R cells stably expressing Ctrl or ITPKB shRNAs were treated with or without 100 μM TMZ for 48 h. ROS levels were measured using the DCFDA assay, and cell apoptosis was assessed after 500 μM TMZ treatment using the Annexin V-FITC apoptosis kit. Data, from three independent samples, are displayed as mean fold change to control ± SD. **f**, **g** TMZ-resistant glioma cells stably expressing Ctrl or ITPKB shRNA-1 were transiently transfected with the wild-type (WT) and kinase-dead mutant D897N of ITPKB. Following 100 μM TMZ treatment, ROS levels were determined by DCFDA assay, and cell apoptosis was assessed after 500 μM TMZ treatment using the Annexin V-FITC apoptosis kit. **h**, **i** T98G-R cells stably expressing Ctrl or ITPKB shRNAs were treated with 100 μM TMZ and/or 1000 U/ml antioxidant enzyme Catalase. ROS levels were measured using the DCFDA assay, and relative cell survival was determined by CCK8 assay. Error bars represent ± SD from three independent experiments. **j** T98G-R cells stably expressing Ctrl or ITPKB shRNAs were treated with or without 100 μM TMZ for 48 h, and NADH Oxidase Activity Assay Kit was used to analyze NOX activity. **k**, **l** T98G-R cells stably expressing Ctrl or ITPKB shRNAs were treated with 100 μM TMZ and/or 10 μM NOX1/4 inhibitor GKT137831. ROS was measured using the DCFDA assay. For cell apoptosis, T98G-R cells were treated with 500 μM TMZ and/or 10 μM NOX1/4 inhibitor GKT137831, and assessed by the Annexin V-FITC apoptosis kit. **m** The level of IP4 was measured in T98G-R cells stably expressing control or ITPKB shRNAs using an ELISA assay. Statistical significance is shown as: *p < 0.01, **p < 0.001

As illustrated in Fig. 3a, b and Supplementary Fig. 3b, c, TMZ-resistant cells exhibited significantly higher basal ROS levels than sensitive glioma cells. Although TMZ treatment increased ROS levels in sensitive cells (T98G and U118), it did not affect resistant cells (T98G-R and U118-R). Knocking down ITPKB in resistant cells restored TMZ-induced ROS production and apoptosis (Fig. 3c–e and Supplementary Fig. 3d–k). Furthermore, the continued expression of ITPKB WT, but not of the kinase-dead ITPKB-DN mutant, in ITPKB-depleted T98G-R, U118-R, and U87-R cells reversed the phenotype to resistance, including decreased ROS production and apoptosis rates (Fig. 3f, g and Supplementary Fig. 3l–r). These findings suggest that the impact of ITPKB on the chemotherapy response in TMZ-resistant glioma cells depends on ROS homeostasis.

To validate this hypothesis, we used catalase, an enzyme detoxifying hydrogen peroxide, to decrease ROS levels in ITPKB-depleted cells. Catalase treatment reversed the cell survival inhibition caused by ITPKB knockdown (Fig. 3h, i and Supplementary Fig. 3s, t). The NOX family of NADPH oxidases is a key enzyme responsible for ROS production and critical determinants of chemosensitivity in multiple cancers.^32,33^ In T98G-R and U118-R cells, ITPKB depletion significantly induced NOX activity after TMZ treatment (Fig. 3j and Supplementary Fig. 3u). Furthermore, the elevated ROS level was reduced, and apoptosis induced by stable ITPKB knockdown was rescued when the NOX1/4 inhibitor GKT137831 was used to inhibit NOX activity (Fig. 3k, l and Supplementary Fig. 3v, w). Previous studies have reported that the ITPKB product, IP4, can effectively inhibit NOX4 activity in cisplatin-resistant cancer cells.^20^ In TMZ-resistant cells, we observed a significant decrease in IP4 levels after ITPKB knockdown (Fig. 3m and Supplementary Fig. 3x). These data indicate that the downregulated expression of ITPKB increases TMZ sensitivity by inducing NOX activity and ROS accumulation.

### E3 ligase Trim25 is an ITPKB-binding protein

**Fig. 4:**
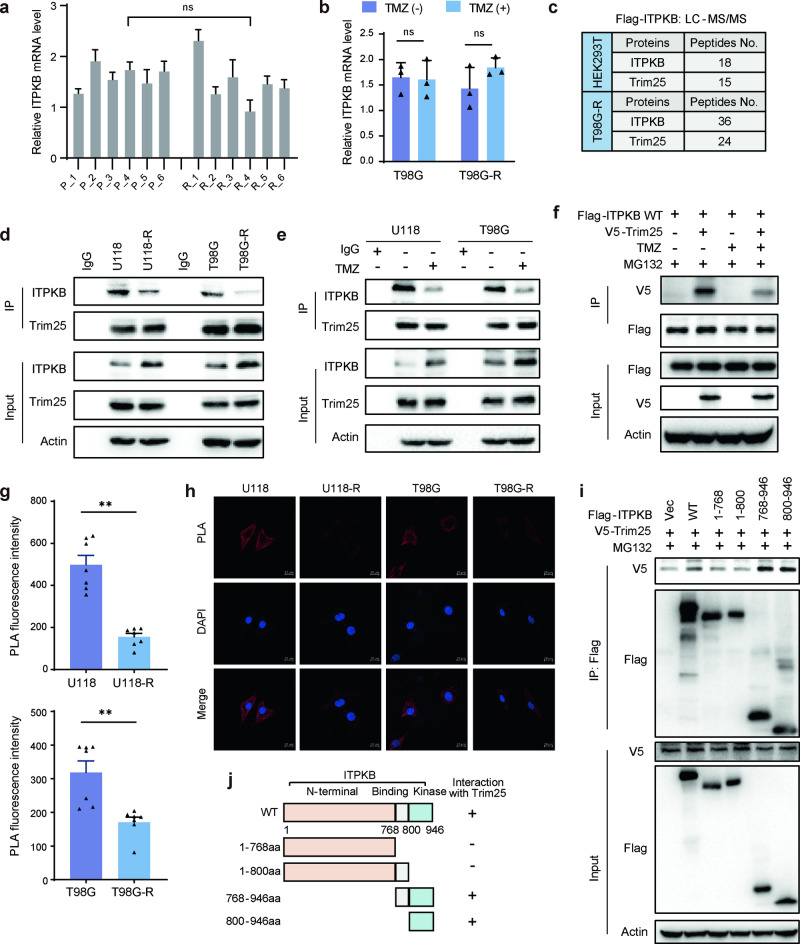
Trim25 is an ITPKB-binding protein. **a** Relative ITPKB mRNA levels in six pairs of GBM tissues from Fig. 1a were determined by RT-PCR. **b** Transcriptional mRNA levels of ITPKB were analyzed in TMZ-sensitive and resistant cells after TMZ treatment by RT-PCR. **c** A list of ITPKB-binding proteins was identified through mass spectrometric analysis. HEK293T and T98G-R cells expressing Flag-ITPKB were generated, and ITPKB complexes were subjected to mass spectrometric analysis. **d** Glioma cell lysates from TMZ-sensitive (T98G, U118) and resistant (T98G-R, U118-R) cells were immunoprecipitated with control IgG or anti-Trim25 antibody, followed by blotting with indicated antibodies. **e** T98G and U118 cells treated with 100 μM TMZ for 48 h had their cell lysates subjected to immunoprecipitation with control IgG or anti-Trim25 antibody. **f** HEK293T cells expressing wild-type Flag-ITPKB were transfected with V5-tagged Trim25. Cells were pretreated with 100 μM TMZ, then co-treated with 50 μM MG132 for an additional 3 h. Cell lysates were immunoprecipitated with Anti-FLAG® M2 Magnetic Beads, followed by blotting with the indicated antibodies. **g**, **h** Representative images of merged Proximity Ligation Assay (PLA) and nuclei (DAPI) channels from PLA experiments. In situ PLA was utilized to assess the interaction between Trim25 and endogenous ITPKB in TMZ-sensitive and resistant glioma cells. Each red dot represents the detection of the Trim25-ITPKB interaction complex, and the graphs representing mean ± SD are shown in (**g**). The scale bar in the bottom left is 20 μm. **i**, **j** Trim25 was found to bind to the kinase domain of ITPKB. HEK293T cells were transiently transfected with V5-Trim25 along with wildtype (WT) and truncated mutants (1–768, 1–800, 768–946, and 800–946 aa) of Flag-ITPKB. The protein interaction was assayed by immunoprecipitation with Anti-FLAG® M2 Magnetic Beads, followed by blotting with the indicated antibodies. Schematic representation of Flag-ITPKB and its deletion mutants is shown in (**j**)

Given the observed increase in ITPKB protein levels, but not mRNA levels, in six pairs of primary and recurrent tissues (Supplementary Fig. 1b and Fig. 4a) and the finding that TMZ induction did not alter ITPKB mRNA levels (Fig. 4b and Supplementary Fig. 4a), we hypothesized that the elevated ITPKB protein level in TMZ-resistant cells occurred through a posttranscriptional mechanism. To verify this, we conducted coimmunoprecipitation and mass spectrometry experiments after overexpressing Flag-tagged ITPKB in HEK293T and T98G-R cells. Among the three main potential binding partners, AP2A1 or CLIP1 was not related to ITPKB stability, as evidenced by data showing that AP2A1 or CLIP1 knockdown did not affect ITPKB levels (Supplementary Fig. 4b–d). Our focus then turned to Trim25, and we validated its binding to ITPKB in HEK293T and T98G-R cells (Fig. 4c). Immunoprecipitation assays using a Trim25 antibody confirmed the interaction between Trim25 and endogenous ITPKB in glioma cells, with a weaker interaction observed in resistant cell lines compared to sensitive cells (Fig. 4d).

The binding ability of ITPKB and Trim25 decreased after TMZ treatment in T98G and U118 cells (Fig. 4e). Furthermore, V5-tagged Trim25 was detected in immunoprecipitation assays using anti-Flag magnetic beads from cells expressing Flag-ITPKB, and treatment with TMZ reduced the interaction between Trim25 and ITPKB (Fig. 4f). Further confirmation of the close interaction between Trim25 and ITPKB was obtained by proximity ligation assay (PLA), revealing more dots representing the interaction between Trim25 and ITPKB in TMZ-sensitive cells than in resistant cells (Fig. 4g–h). To identify the region(s) of ITPKB that mediates its interaction with Trim25, we constructed a series of Flag-tagged ITPKB truncation mutants. Co-IP analysis showed that the ITPKB kinase domain was sufficient and necessary for its binding to Trim25 (Fig. 4i–j). Overall, these results suggest that Trim25 interacts with ITPKB in cells, and this interaction is related to the cellular response to TMZ.

### Trim25 mediates K48-linked ubiquitination of ITPKB at K793 and K818 sites

**Fig. 5:**
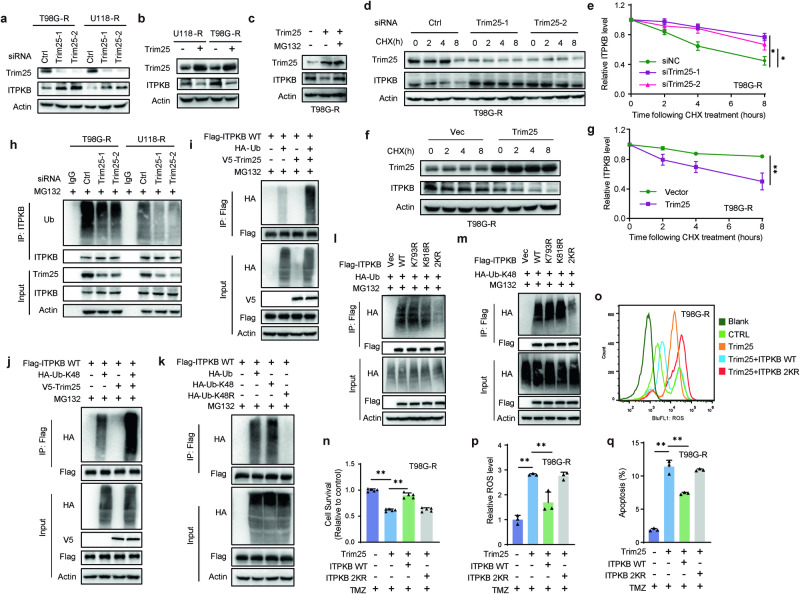
ITPKB interaction with Trim25 dependent on K48 ubiquitination at sites K793 and K818. **a** Depletion of Trim25 induces ITPKB protein levels. T98G-R and U118-R cells were transfected with control (Ctrl) or Trim25 siRNAs, and cell lysates were immunoblotted with the indicated antibodies. **b** T98G-R and U118-R cells were transiently transfected with Trim25 plasmid, and the cell lysates were immunoblotted with the indicated antibodies. **c** T98G-R cells transfected with Ctrl or Trim25 plasmid were treated with vehicle or MG132 (50 μM) for 3 h. Cell lysates were then immunoblotted with the indicated antibodies. **d** T98G-R cells transfected with Ctrl or Trim25 siRNAs were treated with CHX (0.1 mg/mL) and harvested at the indicated times. Cells were lysed, and cell lysates were then immunoblotted with the indicated antibodies. **e** Quantification of the ITPKB protein levels relative to Actin. **f** T98G-R cells transfected with Trim25 plasmid were treated with CHX (0.1 mg/mL) and harvested at the indicated times. **g** Quantification of the ITPKB protein levels relative to Actin. **h** Cells transfected with Ctrl or Trim25 siRNAs were treated with MG132 for 3 h before harvest. Immunoprecipitation with control IgG and ITPKB was performed, followed by immunoblotting with the indicated antibodies. **i**, **j** HEK293T cells expressing Flag-ITPKB were transiently transfected with V5-tagged Trim25 and HA-tagged ubiquitin/K48 ubiquitin. After 48 h, cells were treated with MG132 (50 μM) for 3 h. Cell lysates were immunoprecipitated with Anti-FLAG® M2 Magnetic Beads, and then immunoblotted with the indicated antibodies. **k** HEK293T cells expressing Flag-ITPKB were transiently transfected with indicated HA-K48 lysine-specific mutant constructs. After 48 h, cells were treated with MG132 for 3 h before collection. Cell lysates were immunoprecipitated and immunoblotted with the indicated antibody. **l**, **m** Identification of the ubiquitination sites of ITPKB for its K48-specific polyubiquitination. ITPKB stably knockdown HEK293T cells were transiently transfected with indicated constructs. After 48 h, cells were treated with MG132 for 3 h before collection. Cell lysates were immunoprecipitated and then immunoblotted with the indicated antibodies. **n**–**q** Trim25 plasmids were co-transfected with a control vector, ITPKB wildtype, or 2KR mutant in T98G-R cells. ROS levels were measured using the DCFDA assay. Cell apoptosis was assessed by the Annexin V-FITC apoptosis kit. Relative cell survival was determined by CCK8 assay. Statistical significance is shown as: *p < 0.01, **p < 0.001

To explore the biological significance of the interaction between Trim25 and ITPKB in glioma cells, we further examined cellular responses upon loss or gain of Trim25 function. In T98G-R and U118-R cells, the knockdown of Trim25 by two different siRNAs increased the level of ITPKB (Fig. 5a). At the same time, Trim25 overexpression reduced the ITPKB protein level (Fig. 5b). The decreased ITPKB level after Trim25 overexpression was reversed by adding MG132, a proteasome inhibitor, indicating that Trim25 reduces ITPKB stability through the proteasome pathway (Fig. 5c and Supplementary Fig. 5a). We performed cycloheximide (CHX) chase assays to determine the protein stability of ITPKB. We found that Trim25 knockdown increased the half-life of the ITPKB protein in both T98G-R and U118-R cells (Fig. 5d, e and Supplementary Fig. 5b, c). In contrast, ITPKB stability was significantly attenuated in glioma cells with Trim25 overexpression (Fig. 5f, g and Supplementary Fig. 5d, e).

To test whether Trim25-mediated ubiquitination of ITPKB caused a change in ITPKB stability, ubiquitination assays were performed under various conditions. Trim25 knockdown in T98G-R and U118-R cells significantly decreased the endogenous polyubiquitination of ITPKB compared to control cells (Ctrl) (Fig. 5h). Trim25 overexpression increased ITPKB polyubiquitination in HEK293T cells expressing Flag-tagged ITPKB (Fig. 5i). To further elucidate the characteristics of Trim25-mediated ITPKB ubiquitination, we determined the types of ubiquitin linkage using K48-specific and K63-specific ubiquitin plasmids in which only one specific lysine was retained. The ubiquitination assays revealed that ITPKB was extensively ubiquitinated with Ub-K48 but not Ub-K63 (Fig. 5j and Supplementary Fig. 5f). Using Ub-K48 and its mutant Ub-K48R, we found that the K48 polyubiquitination of ITPKB was abolished by the Ub-K48R mutation (Fig. 5k). To examine which lysine residues in ITPKB were ubiquitinated by Trim25, we used BioGRID (https://thebiogrid.org)^34^ and the web-based GPS-Uber databases (http://gpsuber.biocuckoo.cn/online.php),^35^ predicting that ITPKB K793 and K818 might be potential ubiquitination sites near their binding domain with Trim25. The ITPKB K793R and K818R mutants were then generated, and the single-site mutation (K793R or K818R) showed little effect on polyubiquitination, while the two K to R mutations (2KR) almost completely abolished K48-linked polyubiquitination in ITPKB (Fig. 5l–m). To further verify whether the Trim25-ITPKB-ROS target axis is associated with TMZ resistance, the Trim25 plasmid was co-transfected with a control vector, wild-type ITPKB, or 2KR mutant plasmids in TMZ-resistant GBM cells. In Trim25 overexpression, the ITPKB wildtype, but not the 2KR mutant, increased the survival of T98G-R and U118-R cells (Fig. 5n and Supplementary Fig. 5g, h), accompanied by decreased ROS levels (Fig. 5o–p and Supplementary Fig. 5i–k) and a reduced rate of apoptosis (Fig. 5q and Supplementary Fig. 5l, m). These data demonstrate that the interaction between ITPKB and Trim25 leads to the K48 polyubiquitination of ITPKB, decreasing ITPKB stability.

**Fig. 6:**
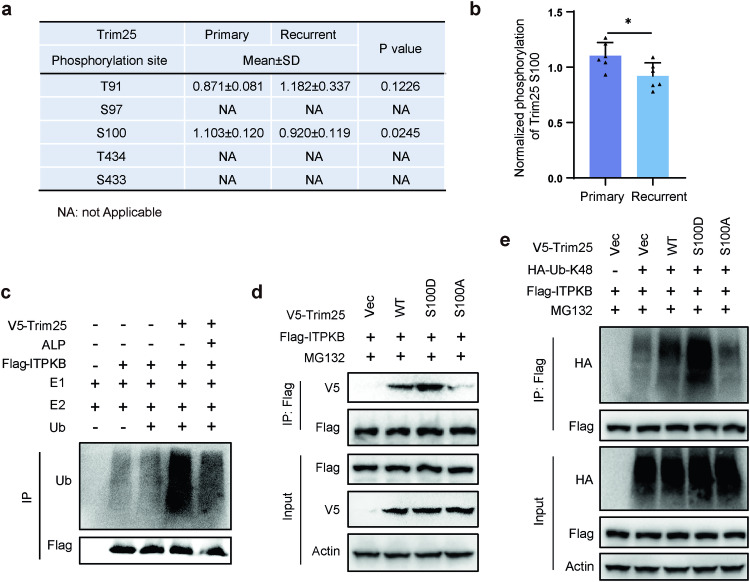
Phosphorylation of Trim25 at S100 is required for ITPKB ubiquitination. **a** Phosphorylation proteomics analysis of Trim25 in primary and recurrence GBM. **b** The phosphorylation level of Trim25 S100 site in GBM tissue. **c** Dephosphorylation of the E3 ligase Trim25 resulted in a more pronounced alleviation of ITPKB ubiquitination in a cell-free system compared to mock-treated Trim25. Dephosphorylated or mock-treated Trim25 was incubated with immunopurified Flag-ITPKB, ubiquitin, recombinant E1 (Uba1), and E2 (UbcH5b). ITPKB ubiquitination was then determined using a ubiquitination assay. **d** The interaction between ITPKB and Trim25 was affected by the Trim25 S100D phosphomimetic mutant and S100A dephosphorylation mutant. **e** The ubiquitination of ITPKB was altered when the S100 site was mutated to Asp (S100D) or Ala (S100A). HEK293T cells were co-transfected with the respective plasmids and treated with MG132 for 3 h after 48 h of transfection. Cell lysates were immunoprecipitated and blotted with specific antibodies for analysis. Statistical significance is shown as: *p < 0.01

To investigate the reasons for the functional differences of Trim25 in primary and recurrent GBM, we analyzed quantitative proteomic data, showing that there was no difference in Trim25 level between primary and recurrent samples (Supplementary Fig. 6a). Since post-translational modifications, such as phosphorylation, have been shown to play an important role in modulating Trim25 activities,^26,36,37^ we performed a phosphoproteomics analysis of Trim25 phosphorylation in the six pairs of samples from primary and recurrent GBM tumors. Among the five potential phosphorylation sites in Trim25, only phosphorylation at the S100 site was significantly reduced in the recurrent samples (Fig. 6a, b), suggesting that the decreased level of S100 phosphorylation in Trim25 might disrupt the activity of the Trim25 E3 ligase, leading to increased stability of ITPKB. To test this hypothesis, in vitro ubiquitination assays were conducted and showed that ITPKB ubiquitination was significantly alleviated when Trim25 was dephosphorylated by alkaline phosphatase (ALP) (Fig. 6c). Furthermore, through phosphomimetic and dephosphorylation approaches, we found that the binding ability of Trim25 to ITPKB was enhanced in the Trim25 S100D mutation (which mimics a phosphomimetic form) but reduced in the Trim25 S100A mutation (which mimics a dephosphorylated form) (Fig. 6d). In contrast, the Trim25 S100D mutant further increased the ubiquitination level of ITPKB, while the Trim25 S100A mutant decreased the ubiquitination level of ITPKB compared to wild-type Trim25 (Fig. 6e). These observations suggest that phosphorylation of the S100 site is necessary for Trim25-mediated ubiquitination of the ITPKB protein.

### ITPKB deficiency increased TMZ sensitivity in vivo

**Fig. 7:**
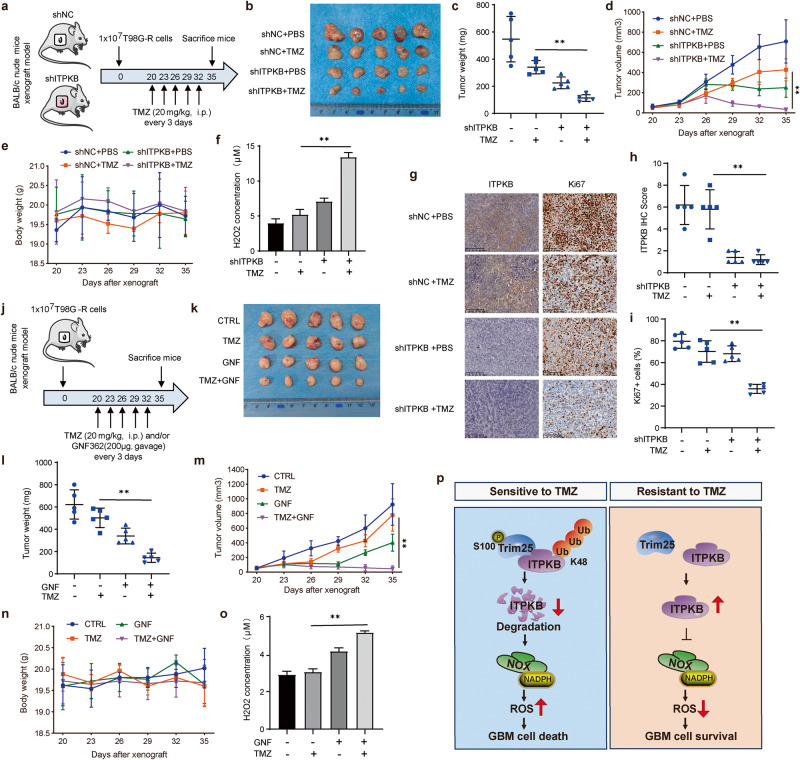
In vivo regulation of glioma TMZ sensitivity by ITPKB through ROS homeostasis*.* **a** Schematic diagram of tumor xenograft experiments using ITPKB knockdown glioma cells. 1 × 10^7^ cells were subcutaneously injected into nude mice. Tumor volumes were measured at indicated days. Mice were sacrificed after 5 weeks. **b** Tumor images were acquired (*n* = 5). **c** Tumor weights were measured and represented as mean tumor weight ± SD. **d**, **e** Tumor volume and mice body weight measured on the indicated day, represented as mean ± SD. **f** Tissues from **a** were homogenized and centrifuged. The H_2_O_2_ concentration of each tissue was analyzed by the H_2_O_2_ assay kit at 560 nm absorbance. **g** Representative IHC staining image of tissues from **a** using ITPKB and Ki67 antibodies. **h**, **i** Quantifications of ITPKB and Ki67 IHC results. **j** Schematic diagram of tumor xenograft experiments using combined treatment with GNF362 and TMZ. Tumor images of each group were shown in **k**, and tumor weight represented as mean ± SD was shown in (**l**). **m**, **n** Tumor volume and mice body weight measured on the indicated day represented as mean ± SD. **o** Tissues from **j** were homogenized and centrifuged. The H_2_O_2_ concertation of each tissue was analyzed by the H_2_O_2_ assay kit at 560 nm absorbance. **p** A schematic representation of ITPKB degradation by E3 ligase Trim25, participating in TMZ resistance of glioma through ROS homeostasis. Statistical significance is indicated as: **p < 0.001

To investigate the relevance of ITPKB to TMZ resistance in vivo, we subcutaneously implanted ITPKB-depleted T98G-R cells in nude mice. We monitored tumor growth for five weeks (Fig. 7a). Thirty-five days after tumor cell implantation, we observed that ITPKB knockdown, in combination with TMZ treatment, significantly decreased tumor growth and tumor weight compared to the control group (Fig. 7b–d). The mice’s body weight did not change significantly during the experiment (Fig. 7e). To validate the impact of ITPKB on ROS homeostasis in vivo, we assayed H_2_O_2_ concentration in tumors from in vivo tumorigenesis experiments. The H_2_O_2_ level increased dramatically in the ITPKB-depleted and TMZ-treated samples (Fig. 7f). The expression of ITPKB and the proliferative marker Ki67 in the above experiment was validated by IHC staining. The results indicated that Ki67 staining decreased in the ITPKB knockdown groups treated with TMZ (Fig. 7g–i).

To further confirm the tumor-promoting function of ITPKB in TMZ-resistant glioma, we examined tumor growth in vivo after treatment with the ITPKB inhibitor GNF362 (Fig. 7j). After 15 days of TMZ and/or GNF362 treatment, the combined treatment group exhibited a significantly better therapeutic response in xenograft tumors. Throughout the experiment, mice receiving combination treatment showed an expected decrease in tumor growth with no change in body weight (Fig. 7k–n). Additionally, the combination treatment group showed a significantly increased H_2_O_2_ level similar to that of ITPKB depletion (Fig. 7o). These data support the notion that ITPKB plays a vital role in regulating the chemotherapy response of TMZ-resistant glioma through ROS homeostasis.

## Discussion

TMZ serves as the primary chemotherapeutic agent for GBM treatment; however, intrinsic or acquired resistance to TMZ significantly limits the efficacy of the drug.^38^ Numerous combinations of TMZ with various chemotherapy, immunotherapy, and radiotherapy methods have been investigated to overcome the challenges in GBM treatment.^39,40^ Despite these efforts, clinically significant benefits from such combinations have yet to be realized, underscoring the need for more understanding of TMZ resistance mechanisms to achieve successful GBM treatment.^41^ This study elucidates a novel mechanism of TMZ resistance, in which ubiquitously expressed lipid kinase ITPKB in recurrent GBM influences TMZ sensitivity in glioma by modulating ROS homeostasis. These findings suggest that ITPKB is a potential therapeutic target for TMZ resistance.

The exploration of GBM development has predominantly focused on identifying biomarkers and novel drug targets for drug resistance. The current study identified four genes that exhibit differential expression in mixed samples obtained from six pairs of primary and recurrent patients. In particular, only ITPK1 and ITPKB were validated in the paired samples. Previous research has indicated that ITPK1 can inhibit TNF-induced apoptosis by interfering with the activation of the TNF-R1-associated death domain.^42^ Consistent with this finding, our study reveals an elevated expression of ITPK1 in recurrent GBM samples (Fig. 1f). However, the pathological significance of increased ITPK1 expression in recurrent GBM remains to be elucidated.

The tripartite motif protein family constitutes a subfamily of RING domain-containing proteins characterized by a cluster of a RING domain, zinc-finger domains, a B box/coiled-coil domain, and a C-terminal SPRY domain. These proteins demonstrate E3 ubiquitin ligase activity along with other non-E3 ubiquitin ligase activities.^43^ Among them, Trim25, an E3 ubiquitin ligase, plays a role in various physiological and pathological phenotypes, including cancer, development, and innate immunity.^44^ Trim25 has been reported to mediate K48- and K63-linked ubiquitination, making it a necessary and sufficient regulator for key cell events.^45–47^ In lung cancer, Trim25 binds to PTEN, facilitating its K63-linked ubiquitination. This interaction prevents the translocation of PTEN to the plasma membrane, reducing its phosphatase activity and consequently activating the AKT/mTOR pathway.^48^ In mammalian cells, Trim25 interacts with the N-terminal caspase recruitment domains (CARDs) of the retinoic acid-inducible gene (RIG-I), inducing Lys 63-linked ubiquitination of RIG-I, leading to a significant increase in cytosolic RIG-I activity.^49^ Trim25, an estrogen-responsive finger protein, has been reported to target the negative cell cycle regulator 14-3-3σ by mediating its K48-linked ubiquitination and subsequent degradation.^50^ Furthermore, cyclophilin A competitively binds to MAVS, inhibiting the interaction of Trim25 with MAVS and preventing Trim25-induced K48-linked ubiquitination and proteasomal degradation of MAVS.^51^ Based on previous findings, our study unveils ITPKB as another Trim25 substrate. Trim25 mediates the K48-ubiquitination of ITPKB, a crucial regulator of ROS homeostasis, leading to degradation of ITPKB and upregulation of NOX activity in the presence of TMZ in GBM cells.

Therapeutic approaches centered on kinase inhibitors, particularly receptor tyrosine kinase inhibitors, have demonstrated unique advantages in overcoming drug resistance in glioblastoma.^52^ In particular, the T-cell activation-related protein ITPKB has emerged as a potential therapeutic target for managing graft-versus-host disease (GVHD) in clinical settings.^53^ Therapeutic administration of the ITPKB inhibitor GNF362 has shown efficacy in alleviating GVHD by selectively eliminating donor alloreactive T cells compared to nominal antigen-responsive T cells and reducing lung immunoglobulin deposition.^17^ ITPKB signaling is crucial to provide a metabolic advantage and confer cisplatin resistance in various human cancers, including head and neck squamous cell carcinoma, lung cancer, and ovarian cancer.^20^ However, limited reports link the application of the orally active ITPKB inhibitor GNF362 to solid human tumors. For the first time, we report that selective inhibition of ITPKB by GNF362 significantly hinders tumor progression in a TMZ-resistant mouse model. This revelation unveils a novel role for the ITPKB pathway in TMZ-resistant glioblastoma. It underscores the potential utility of GNF362 to be an adjuvant therapy for treating recurrent GBM in clinical settings.

In conclusion, although previous research has elucidated the role of ITPKB in cisplatin resistance through the NOX4-redox mechanism, pivotal questions persist: How is ITPKB sustained at elevated levels in drug-resistant cells? Is the underlying mechanism specific to particular drugs? Can ITPKB be a viable target to mitigate drug resistance? Our study seeks to address these inquiries. We demonstrate that ITPKB is highly expressed in TMZ-resistant GBM cells, implying its potential significance as a pivotal regulator in drug resistance. Using clinically informative paired samples, Trim25 is identified as an E3 ligase responsible for modulating ITPKB stability, elucidating a novel ITPKB-NOX-ROS signaling axis that orchestrates drug resistance (Fig. 7p). Applying GNF362 to target ITPKB has enhanced the sensitivity of resistant GBM tumors to TMZ.

### Supplementary information

Supplementary information
