# Supplementary material for: Suppression of ITPKB degradation by Trim25 confers TMZ resistance in glioblastoma through ROS homeostasis
Source: Signal Transduct Target Ther. 2024 Mar 4;9:58. doi: 10.1038/s41392-024-01763-x (PMC10912509; doi:10.1038/s41392-024-01763-x)

Supplementary Materials for

Suppression of ITPKB degradation by Trim25 confers TMZ resistance in glioblastoma through ROS homeostasis

Yuanliang Yan^1^, Shangjun Zhou^1^, Xi Chen^1^, Qiaoli Yi^1^, Songshan Feng^2^, Zijin Zhao^2^, Yuanhong Liu^1^, Qiuju Liang^1^, Zhijie Xu^3^*, Zhi Li^4,5,6^*, Lunquan Sun^4,5,6^*

^1^ Department of Pharmacy, Xiangya Hospital, Central South University, Changsha 410008, China.^2^ Department of Neurosurgery, Xiangya Hospital, Central South University, Changsha 410008, China.^3^ Department of Pathology, Xiangya Hospital, Central South University, Changsha 410008, China. ^4^ Xiangya Cancer Center, Xiangya Hospital, Central South University, Changsha 410008, China; ^5^ Key Laboratory of Molecular Radiation Oncology Hunan Province, Changsha 410008, China; ^6^ Institute of Cancer Research, National Clinical Research Center for Geriatric Disorders (Xiangya), Xiangya Hospital, Central South University, Changsha 410008, China.

Correspondence to: Zhijie Xu (xzj1322007@csu.edu.cn) or Zhi Li (peries@csu.edu.cn) or Lunquan Sun (lunquansun@csu.edu.cn)

**This PDF file includes:**

Tables S1

Figures. S1 to S6

TABLE OF CONTENTS

Supplementary table 1. Differential expression analysis of 648 proteins between primary and recurrent GBM patients1

Supplementary Figure S1. Expression profiles of ITPKB in glioma6

Supplementary Figure S2. ITPKB mediates TMZ sensitivity in GBM cells through its kinase activity7

Supplementary Figure S3. ITPKB involvement in TMZ sensitivity through ROS homeostasis8

Supplementary Figure S4. Trim25 is an ITPKB binding protein10

Supplementary Figure S5. ITPKB interaction with Trim25 depends on its K48 ubiquitination at sites K793 and K81810

Supplementary Figure S6. Trim25 protein levels in primary and recurrent GBM patients11

**Supplementary table 1. Differential expression analysis of 648 proteins between primary and recurrent GBM patients.**

| Protein Name | Fold Change | P Value | Protein Name | Fold Change | P Value | Protein Name | Fold Change | P Value |
| --- | --- | --- | --- | --- | --- | --- | --- | --- |
| PLP1 | 3.5038 | 0.0344 | PADI2 | 2.0276 | 0.0045 | EIF1AY | 1.6921 | 0.0072 |
| FTL | 3.3904 | 0.0377 | MAOB | 2.0138 | 0.0076 | RHOG | 1.6881 | 0.0015 |
| MBP | 3.3741 | 0.0390 | CAPG | 2.0089 | 0.0256 | ELOVL1 | 1.6854 | 0.0193 |
| CLDN11 | 3.0824 | 0.0387 | NQO1 | 1.9853 | 0.0167 | IGHV4-28 | 1.6844 | 0.0296 |
| FOLR3 | 3.0646 | 0.0207 | HSPB8 | 1.9777 | 0.0079 | SELENBP1 | 1.6767 | 0.0029 |
| MOG | 2.9714 | 0.0333 | FTH1 | 1.9763 | 0.0031 | NDRG1 | 1.6678 | 0.0244 |
| GLDN | 2.8255 | 0.0478 | FBXO2 | 1.9700 | 0.0143 | PLEKHB1 | 1.6650 | 0.0311 |
| EPHA3 | 2.7858 | 0.0056 | TPPP3 | 1.9535 | 0.0015 | AMPD3 | 1.6538 | 0.0055 |
| PPP1R14A | 2.7585 | 0.0141 | PIR | 1.9475 | 0.0000 | INF2 | 1.6402 | 0.0019 |
| MAG | 2.7109 | 0.0458 | LIPA | 1.9466 | 0.0293 | PBXIP1 | 1.6270 | 0.0004 |
| GPNMB | 2.6598 | 0.0293 | CMTM5 | 1.9384 | 0.0442 | CSTB | 1.6262 | 0.0227 |
| FABP5 | 2.6327 | 0.0072 | FA2H | 1.9331 | 0.0465 | QPRT | 1.6224 | 0.0018 |
| S100A1 | 2.6157 | 0.0062 | FOLH1 | 1.9288 | 0.0220 | AK1 | 1.6073 | 0.0143 |
| HSPA2 | 2.6015 | 0.0069 | RENBP | 1.9025 | 0.0030 | BAG3 | 1.6011 | 0.0015 |
| GJC2 | 2.6011 | 0.0152 | S100A6 | 1.8756 | 0.0269 | GRIK3 | 1.5977 | 0.0464 |
| CARNS1 | 2.6001 | 0.0133 | CYP27A1 | 1.8427 | 0.0364 | ALAD | 1.5956 | 0.0011 |
| ANLN | 2.5977 | 0.0332 | S100B | 1.8395 | 0.0056 | OSTF1 | 1.5941 | 0.0446 |
| SYNM | 2.4989 | 0.0021 | GLTP | 1.8388 | 0.0219 | CD109 | 1.5928 | 0.0126 |
| CD9 | 2.4405 | 0.0230 | CTSD | 1.8285 | 0.0241 | UGT8 | 1.5920 | 0.0314 |
| SIRT2 | 2.4263 | 0.0461 | S100A13 | 1.8261 | 0.0042 | HSPB6 | 1.5760 | 0.0140 |
| MYO1D | 2.3480 | 0.0153 | CYB5R2 | 1.8093 | 0.0121 | ELMO1 | 1.5707 | 0.0028 |
| GPD1 | 2.3240 | 0.0421 | TPD52 | 1.7942 | 0.0394 | HRG | 1.5650 | 0.0315 |
| EFHD1 | 2.3051 | 0.0100 | EPHX1 | 1.7921 | 0.0002 | ANXA11 | 1.5625 | 0.0048 |
| GJB1 | 2.2452 | 0.0445 | CAB39L | 1.7841 | 0.0117 | LGALS1 | 1.5540 | 0.0305 |
| FOLR2 | 2.2414 | 0.0016 | PLLP | 1.7832 | 0.0211 | NEBL | 1.5522 | 0.0230 |
| ERMN | 2.2175 | 0.0257 | SYNGR2 | 1.7829 | 0.0070 | ADSSL1 | 1.5462 | 0.0461 |
| ALDH1A1 | 2.2017 | 0.0001 | MGST1 | 1.7739 | 0.0128 | RBKS | 1.5400 | 0.0006 |
| PMP2 | 2.1957 | 0.0307 | LITAF | 1.7732 | 0.0215 | SHTN1 | 1.5376 | 0.0279 |
| CAPS | 2.1812 | 0.0117 | CLDND1 | 1.7547 | 0.0334 | HEBP1 | 1.5345 | 0.0026 |
| ENPP6 | 2.1675 | 0.0408 | SLC44A1 | 1.7459 | 0.0259 | APOD | 1.5329 | 0.0355 |
| NINJ2 | 2.1492 | 0.0162 | MAP7 | 1.7373 | 0.0218 | NENF | 1.5319 | 0.0007 |
| CPM | 2.1365 | 0.0005 | PTGDS | 1.7248 | 0.0081 | ITPRID2 | 1.5289 | 0.0453 |
| LACC1 | 2.1229 | 0.0082 | CDK18 | 1.7193 | 0.0374 | DOCK5 | 1.5263 | 0.0213 |
| SEPT4 | 2.1180 | 0.0346 | JAM3 | 1.7169 | 0.0123 | CD59 | 1.5221 | 0.0009 |
| BCAS1 | 2.0978 | 0.0325 | TPP1 | 1.7088 | 0.0066 | ITGA2 | 1.5203 | 0.0341 |
| ADIRF | 2.0361 | 0.0324 | ABCA2 | 1.7003 | 0.0046 | RASAL3 | 1.5165 | 0.0060 |
| HTATIP2 | 2.0302 | 0.0192 | CBR1 | 1.7000 | 0.0085 | ZFYVE19 | 1.5164 | 0.0053 |
| CPPED1 | 2.0290 | 0.0001 | PIP4K2A | 1.6939 | 0.0140 | C1orf198 | 1.5132 | 0.0149 |
| NT5DC1 | 1.5081 | 0.0015 | FGF1 | 1.4174 | 0.0001 | KIAA0930 | 1.3424 | 0.0048 |
| GMPR | 1.5032 | 0.0140 | MAPK11 | 1.4119 | 0.0008 | SOD1 | 1.3417 | 0.0375 |
| PLIN3 | 1.5030 | 0.0245 | TMBIM1 | 1.4087 | 0.0470 | SMPD1 | 1.3395 | 0.0181 |
| LHPP | 1.5023 | 0.0145 | STAT6 | 1.4073 | 0.0280 | GYG1 | 1.3371 | 0.0123 |
| SELENOM | 1.5021 | 0.0186 | CA2 | 1.4069 | 0.0202 | FAM49B | 1.3362 | 0.0003 |
| PRDX6 | 1.4992 | 0.0264 | TTYH1 | 1.4016 | 0.0326 | PXK | 1.3291 | 0.0114 |
| RAB32 | 1.4978 | 0.0441 | OTUD7B | 1.4008 | 0.0028 | SORL1 | 1.3286 | 0.0373 |
| CIB1 | 1.4972 | 0.0018 | FHIT | 1.4005 | 0.0488 | EPB41L2 | 1.3266 | 0.0371 |
| THSD7B | 1.4967 | 0.0186 | MGLL | 1.3926 | 0.0157 | PRKG1 | 1.3262 | 0.0041 |
| PRDX1 | 1.4894 | 0.0099 | BOP1 | 1.3920 | 0.0004 | HSPA1B | 1.3250 | 0.0484 |
| ENDOD1 | 1.4786 | 0.0005 | APOA2 | 1.3917 | 0.0283 | DNAJB4 | 1.3242 | 0.0151 |
| FAH | 1.4755 | 0.0112 | APOA1 | 1.3899 | 0.0228 | SYPL1 | 1.3195 | 0.0024 |
| RAP1A | 1.4746 | 0.0003 | PON1 | 1.3840 | 0.0160 | MRI1 | 1.3151 | 0.0126 |
| HPX | 1.4744 | 0.0062 | CYBRD1 | 1.3798 | 0.0297 | CD82 | 1.3133 | 0.0288 |
| ANO6 | 1.4733 | 0.0035 | TSR2 | 1.3788 | 0.0113 | PLEKHO2 | 1.3095 | 0.0443 |
| ADD3 | 1.4710 | 0.0230 | KCNAB2 | 1.3728 | 0.0114 | MTM1 | 1.3087 | 0.0131 |
| FLNB | 1.4695 | 0.0249 | AFM | 1.3718 | 0.0396 | REXO2 | 1.3079 | 0.0142 |
| TEX2 | 1.4670 | 0.0003 | LLGL1 | 1.3707 | 0.0395 | ITPKB | 1.3067 | 0.0105 |
| SWAP70 | 1.4654 | 0.0398 | NRBP2 | 1.3688 | 0.0019 | LAMP2 | 1.3060 | 0.0260 |
| ASS1 | 1.4654 | 0.0044 | TMUB1 | 1.3667 | 0.0176 | PACSIN3 | 1.3056 | 0.0309 |
| EPHX2 | 1.4611 | 0.0142 | FNTB | 1.3666 | 0.0071 | TPD52L2 | 1.3029 | 0.0261 |
| ADAM10 | 1.4601 | 0.0050 | SNAP23 | 1.3666 | 0.0094 | PBLD | 1.2989 | 0.0399 |
| APOA4 | 1.4536 | 0.0186 | PDLIM2 | 1.3664 | 0.0256 | NT5C2 | 1.2969 | 0.0405 |
| MTMR10 | 1.4509 | 0.0346 | ACTG2 | 1.3654 | 0.0223 | MAP4 | 1.2907 | 0.0019 |
| FAM177A1 | 1.4505 | 0.0124 | GSTO1 | 1.3632 | 0.0052 | PTP4A2 | 1.2888 | 0.0384 |
| MPP1 | 1.4489 | 0.0106 | EPS15 | 1.3629 | 0.0095 | CAPN2 | 1.2867 | 0.0011 |
| NAT8L | 1.4474 | 0.0006 | TKT | 1.3591 | 0.0300 | NDE1 | 1.2849 | 0.0447 |
| LIMCH1 | 1.4472 | 0.0315 | CNDP1 | 1.3567 | 0.0342 | S100A16 | 1.2843 | 0.0053 |
| ERMP1 | 1.4446 | 0.0093 | RRAS | 1.3561 | 0.0443 | MRVI1 | 1.2842 | 0.0150 |
| MYH14 | 1.4428 | 0.0016 | PHKG1 | 1.3547 | 0.0460 | C2CD2 | 1.2828 | 0.0113 |
| CAST | 1.4401 | 0.0085 | OPLAH | 1.3520 | 0.0081 | TTR | 1.2826 | 0.0383 |
| SUSD2 | 1.4363 | 0.0220 | RAP2B | 1.3500 | 0.0369 | PHKB | 1.2816 | 0.0112 |
| COLEC12 | 1.4331 | 0.0039 | FPR1 | 1.3492 | 0.0120 | HERC2 | 1.2809 | 0.0014 |
| VAMP3 | 1.4287 | 0.0045 | GLRX | 1.3484 | 0.0172 | FNBP1 | 1.2761 | 0.0023 |
| ECHDC1 | 1.4240 | 0.0005 | SH3KBP1 | 1.3472 | 0.0164 | PKP4 | 1.2695 | 0.0414 |
| CD55 | 1.4213 | 0.0154 | SORBS1 | 1.3468 | 0.0040 | BCL2L1 | 1.2668 | 0.0351 |
| ZNF804A | 1.4186 | 0.0389 | CYSTM1 | 1.3452 | 0.0383 | GNAI2 | 1.2655 | 0.0077 |
| CYP11B2 | 1.4179 | 0.0491 | WFS1 | 1.3440 | 0.0029 | SUMO1 | 1.2653 | 0.0356 |
| RAB4B | 1.2635 | 0.0190 | PANK4 | 0.8298 | 0.0383 | MRPL21 | 0.8095 | 0.0179 |
| PHYKPL | 1.2601 | 0.0431 | HSPD1 | 0.8282 | 0.0243 | PMPCA | 0.8084 | 0.0155 |
| RNH1 | 1.2588 | 0.0076 | USP7 | 0.8276 | 0.0224 | PTGES3 | 0.8081 | 0.0284 |
| ZFYVE16 | 1.2561 | 0.0308 | STAU1 | 0.8270 | 0.0013 | RPL19 | 0.8080 | 0.0222 |
| TPM3 | 1.2510 | 0.0364 | CDC16 | 0.8267 | 0.0053 | DLGAP4 | 0.8076 | 0.0005 |
| CAPNS1 | 1.2485 | 0.0099 | ARHGEF7 | 0.8263 | 0.0266 | FXR2 | 0.8062 | 0.0006 |
| PARP10 | 1.2474 | 0.0351 | G3BP2 | 0.8257 | 0.0114 | FDPS | 0.8061 | 0.0118 |
| C9orf64 | 1.2445 | 0.0254 | MRPL58 | 0.8248 | 0.0246 | LIG1 | 0.8034 | 0.0344 |
| GNG5 | 1.2434 | 0.0438 | C17orf75 | 0.8248 | 0.0272 | NUDT9 | 0.8030 | 0.0104 |
| CNDP2 | 1.2403 | 0.0213 | RPS15A | 0.8242 | 0.0433 | SLIRP | 0.8023 | 0.0413 |
| ITPK1 | 1.2359 | 0.0083 | CAND1 | 0.8241 | 0.0080 | PRKRA | 0.8019 | 0.0028 |
| ERBIN | 1.2338 | 0.0437 | ZNF579 | 0.8228 | 0.0156 | ATP2A2 | 0.8016 | 0.0002 |
| HAPLN4 | 1.2323 | 0.0269 | PPP2R5C | 0.8227 | 0.0244 | PRMT5 | 0.8011 | 0.0022 |
| RALB | 1.2301 | 0.0044 | MGRN1 | 0.8220 | 0.0130 | PCBP2 | 0.8011 | 0.0138 |
| MTSS1 | 1.2281 | 0.0266 | TNPO3 | 0.8217 | 0.0484 | C12orf57 | 0.8006 | 0.0179 |
| PDK2 | 1.2276 | 0.0131 | FAM89B | 0.8217 | 0.0233 | PPP2R5E | 0.8005 | 0.0213 |
| PACS2 | 1.2271 | 0.0318 | SLC3A2 | 0.8214 | 0.0107 | GAN | 0.8000 | 0.0110 |
| RAP2C | 1.2269 | 0.0226 | CTBP1 | 0.8212 | 0.0359 | EXD2 | 0.7999 | 0.0465 |
| RAP2C | 1.2269 | 0.0226 | CTBP1 | 0.8212 | 0.0359 | EXD2 | 0.7999 | 0.0465 |
| ITM2C | 1.2250 | 0.0250 | ETFA | 0.8211 | 0.0353 | RPS18 | 0.7998 | 0.0359 |
| AKR1A1 | 1.2244 | 0.0450 | TXN2 | 0.8203 | 0.0479 | DDX6 | 0.7997 | 0.0075 |
| LACTB2 | 1.2241 | 0.0279 | UBE3A | 0.8200 | 0.0056 | MPP6 | 0.7980 | 0.0163 |
| NDUFAF7 | 1.2228 | 0.0118 | SGTA | 0.8199 | 0.0092 | DDX19A | 0.7979 | 0.0106 |
| PRUNE2 | 1.2197 | 0.0140 | RNF2 | 0.8196 | 0.0492 | CSTF2 | 0.7978 | 0.0473 |
| VPS36 | 1.2185 | 0.0411 | NAA10 | 0.8187 | 0.0187 | SUOX | 0.7978 | 0.0240 |
| CCDC91 | 1.2180 | 0.0043 | BSG | 0.8186 | 0.0268 | NAT14 | 0.7975 | 0.0175 |
| NUDT16 | 1.2175 | 0.0209 | RPL5 | 0.8179 | 0.0154 | ACACA | 0.7972 | 0.0250 |
| SYT11 | 1.2162 | 0.0448 | EGLN1 | 0.8172 | 0.0306 | RPL27A | 0.7968 | 0.0302 |
| HEBP2 | 1.2155 | 0.0024 | RAB3D | 0.8172 | 0.0182 | ARMCX1 | 0.7967 | 0.0411 |
| CDC42 | 1.2149 | 0.0059 | TOMM5 | 0.8171 | 0.0303 | RPS10 | 0.7966 | 0.0251 |
| SLC27A1 | 1.2126 | 0.0249 | RANBP1 | 0.8165 | 0.0287 | LARP4B | 0.7963 | 0.0282 |
| ATG7 | 1.2097 | 0.0316 | MRPL49 | 0.8164 | 0.0095 | RPAP1 | 0.7962 | 0.0110 |
| HS1BP3 | 1.2091 | 0.0367 | USP19 | 0.8161 | 0.0434 | DDX3X | 0.7959 | 0.0164 |
| ANXA7 | 1.2090 | 0.0385 | EIF2S1 | 0.8149 | 0.0276 | CNOT7 | 0.7957 | 0.0035 |
| GXYLT1 | 1.2075 | 0.0444 | CDH6 | 0.8148 | 0.0070 | SLC25A11 | 0.7951 | 0.0232 |
| TIGAR | 1.2030 | 0.0286 | MCAM | 0.8146 | 0.0170 | DRG2 | 0.7943 | 0.0037 |
| RNF170 | 1.2021 | 0.0109 | TOMM22 | 0.8135 | 0.0432 | DDX41 | 0.7927 | 0.0183 |
| SPART | 1.2018 | 0.0283 | SPARCL1 | 0.8124 | 0.0471 | PFAS | 0.7917 | 0.0191 |
| PGGT1B | 1.2015 | 0.0372 | RTCB | 0.8119 | 0.0444 | SLC8A1 | 0.7914 | 0.0321 |
| TOMM40 | 0.7902 | 0.0311 | RPL18 | 0.7660 | 0.0198 | TUBB | 0.7435 | 0.0182 |
| CPE | 0.7902 | 0.0294 | PAICS | 0.7660 | 0.0035 | DDX42 | 0.7433 | 0.0331 |
| HSPA9 | 0.7888 | 0.0162 | RPS19 | 0.7655 | 0.0268 | RPS2 | 0.7429 | 0.0122 |
| SLC7A5 | 0.7883 | 0.0358 | GRPEL1 | 0.7647 | 0.0241 | CCAR2 | 0.7427 | 0.0194 |
| FASN | 0.7883 | 0.0329 | SMG7 | 0.7643 | 0.0468 | RPL27 | 0.7424 | 0.0339 |
| DNAJC19 | 0.7876 | 0.0467 | SFXN1 | 0.7632 | 0.0075 | SNRPN | 0.7423 | 0.0295 |
| SMARCA4 | 0.7841 | 0.0399 | RPS3 | 0.7593 | 0.0199 | TTC38 | 0.7418 | 0.0144 |
| NIPSNAP1 | 0.7841 | 0.0288 | RAN | 0.7592 | 0.0132 | RPL24 | 0.7415 | 0.0110 |
| IGFBP4 | 0.7829 | 0.0478 | RPS16 | 0.7592 | 0.0282 | TCERG1 | 0.7414 | 0.0493 |
| MCCC1 | 0.7823 | 0.0373 | PSME3 | 0.7585 | 0.0167 | MRPS27 | 0.7414 | 0.0260 |
| NAA50 | 0.7801 | 0.0473 | RPS25 | 0.7581 | 0.0223 | HNRNPH2 | 0.7408 | 0.0255 |
| LONP1 | 0.7794 | 0.0293 | THOC1 | 0.7572 | 0.0322 | CELF1 | 0.7384 | 0.0228 |
| ACTL6A | 0.7782 | 0.0279 | GEMIN4 | 0.7569 | 0.0393 | RNMT | 0.7380 | 0.0365 |
| PABPN1 | 0.7782 | 0.0430 | PRPF19 | 0.7567 | 0.0251 | HMGCS1 | 0.7379 | 0.0210 |
| PUM1 | 0.7759 | 0.0130 | UBA52 | 0.7564 | 0.0429 | EIF1AX | 0.7377 | 0.0341 |
| PA2G4 | 0.7757 | 0.0424 | SF3B2 | 0.7555 | 0.0364 | USP10 | 0.7352 | 0.0003 |
| ATXN2L | 0.7756 | 0.0006 | WDR82 | 0.7551 | 0.0245 | RPS27L | 0.7351 | 0.0056 |
| RPL32 | 0.7753 | 0.0223 | SLC25A25 | 0.7550 | 0.0025 | SNRPD3 | 0.7340 | 0.0411 |
| RPL10 | 0.7752 | 0.0390 | TRNT1 | 0.7544 | 0.0117 | TBL1XR1 | 0.7338 | 0.0196 |
| FAM136A | 0.7750 | 0.0343 | TIMM44 | 0.7544 | 0.0080 | RPS11 | 0.7335 | 0.0180 |
| CTR9 | 0.7740 | 0.0483 | CTBP2 | 0.7539 | 0.0414 | PTCD3 | 0.7335 | 0.0296 |
| FXR1 | 0.7735 | 0.0310 | RPS5 | 0.7528 | 0.0124 | RPL31 | 0.7335 | 0.0219 |
| SMARCC2 | 0.7733 | 0.0223 | DDX39B | 0.7518 | 0.0458 | GNPNAT1 | 0.7310 | 0.0149 |
| CCAR1 | 0.7729 | 0.0168 | TP53BP1 | 0.7514 | 0.0142 | GRIA2 | 0.7309 | 0.0164 |
| ABI2 | 0.7727 | 0.0171 | HARS2 | 0.7513 | 0.0182 | ERG28 | 0.7302 | 0.0463 |
| RPS26 | 0.7723 | 0.0130 | PAM16 | 0.7511 | 0.0469 | HNRNPUL1 | 0.7302 | 0.0371 |
| GTPBP1 | 0.7717 | 0.0268 | DBN1 | 0.7497 | 0.0224 | LCN2 | 0.7290 | 0.0475 |
| MRPS30 | 0.7712 | 0.0371 | API5 | 0.7491 | 0.0429 | RPS15 | 0.7280 | 0.0240 |
| PCDH17 | 0.7710 | 0.0070 | BOLA3 | 0.7481 | 0.0417 | SLC39A10 | 0.7239 | 0.0007 |
| SMU1 | 0.7709 | 0.0303 | CPD | 0.7477 | 0.0368 | SMARCB1 | 0.7238 | 0.0299 |
| PAFAH1B3 | 0.7709 | 0.0097 | RPS8 | 0.7476 | 0.0213 | CPSF6 | 0.7230 | 0.0209 |
| NAV1 | 0.7708 | 0.0477 | RPS20 | 0.7475 | 0.0143 | MRPS22 | 0.7227 | 0.0409 |
| UBR7 | 0.7708 | 0.0192 | RPL9 | 0.7472 | 0.0143 | SF3B3 | 0.7223 | 0.0351 |
| RPL38 | 0.7702 | 0.0297 | PPIG | 0.7469 | 0.0209 | HNRNPK | 0.7222 | 0.0222 |
| RAB39B | 0.7698 | 0.0062 | RPL11 | 0.7459 | 0.0216 | FAM98B | 0.7220 | 0.0241 |
| NUFIP2 | 0.7682 | 0.0192 | MRPS31 | 0.7446 | 0.0444 | MYO1B | 0.7207 | 0.0472 |
| RBM12B | 0.7674 | 0.0278 | KHDRBS1 | 0.7439 | 0.0367 | PRKDC | 0.6735 | 0.0154 |
| IPO9 | 0.7664 | 0.0083 | BUB3 | 0.7015 | 0.0140 | NCAN | 0.6730 | 0.0171 |
| RBM12 | 0.7196 | 0.0200 | TRAP1 | 0.6999 | 0.0371 | MTA1 | 0.6726 | 0.0343 |
| ATAD3A | 0.7193 | 0.0249 | RBM14 | 0.6987 | 0.0075 | FADS2 | 0.6725 | 0.0444 |
| GSTCD | 0.7190 | 0.0387 | LUC7L2 | 0.6986 | 0.0385 | DHX9 | 0.6725 | 0.0255 |
| RPL8 | 0.7188 | 0.0113 | ITIH5 | 0.6970 | 0.0439 | SNRNP200 | 0.6714 | 0.0493 |
| SRPK1 | 0.7176 | 0.0427 | HNRNPC | 0.6970 | 0.0274 | DHCR7 | 0.6664 | 0.0099 |
| ALKBH5 | 0.7174 | 0.0250 | SNRNP70 | 0.6966 | 0.0445 | EIF4A3 | 0.6664 | 0.0250 |
| MRPS9 | 0.7167 | 0.0322 | MATR3 | 0.6959 | 0.0103 | SET | 0.6659 | 0.0441 |
| RPL7A | 0.7162 | 0.0171 | UBA2 | 0.6954 | 0.0147 | PGAP1 | 0.6659 | 0.0145 |
| RALY | 0.7158 | 0.0207 | SAP18 | 0.6947 | 0.0345 | SMARCA5 | 0.6654 | 0.0275 |
| SF3A3 | 0.7152 | 0.0272 | AGRN | 0.6940 | 0.0045 | LETM1 | 0.6654 | 0.0018 |
| TCAF1 | 0.7148 | 0.0090 | MRPS17 | 0.6929 | 0.0217 | SRSF3 | 0.6641 | 0.0328 |
| SFPQ | 0.7141 | 0.0339 | DHX30 | 0.6920 | 0.0230 | NID1 | 0.6619 | 0.0293 |
| SARNP | 0.7135 | 0.0318 | RPS24 | 0.6918 | 0.0082 | NAT10 | 0.6613 | 0.0165 |
| PRMT1 | 0.7134 | 0.0174 | C1QBP | 0.6909 | 0.0051 | NCOA5 | 0.6613 | 0.0491 |
| U2SURP | 0.7132 | 0.0374 | ACAD10 | 0.6900 | 0.0469 | SART3 | 0.6609 | 0.0345 |
| ATP2B4 | 0.7132 | 0.0203 | RPS4X | 0.6895 | 0.0062 | SRSF1 | 0.6602 | 0.0423 |
| PTPRZ1 | 0.7125 | 0.0222 | RPL15 | 0.6885 | 0.0116 | SRSF7 | 0.6601 | 0.0334 |
| GNG2 | 0.7122 | 0.0466 | RPL34 | 0.6875 | 0.0036 | U2AF2 | 0.6587 | 0.0266 |
| ATXN10 | 0.7117 | 0.0042 | FARSA | 0.6874 | 0.0043 | HNRNPH1 | 0.6575 | 0.0125 |
| ADAR | 0.7110 | 0.0429 | SMYD3 | 0.6864 | 0.0060 | NARS2 | 0.6573 | 0.0364 |
| PSPC1 | 0.7105 | 0.0022 | SNAP47 | 0.6849 | 0.0378 | TRIM28 | 0.6562 | 0.0221 |
| CDC5L | 0.7102 | 0.0360 | RBBP7 | 0.6842 | 0.0270 | SRRT | 0.6557 | 0.0256 |
| HNRNPDL | 0.7086 | 0.0466 | RPL30 | 0.6840 | 0.0021 | PPM1G | 0.6557 | 0.0131 |
| SLC39A6 | 0.7074 | 0.0153 | LRPPRC | 0.6828 | 0.0047 | DPP6 | 0.6552 | 0.0125 |
| RPL18A | 0.7069 | 0.0334 | RPS9 | 0.6817 | 0.0095 | QRICH1 | 0.6548 | 0.0470 |
| NSMCE3 | 0.7059 | 0.0452 | HIST2H2BE | 0.6817 | 0.0336 | TARDBP | 0.6532 | 0.0150 |
| ILF3 | 0.7059 | 0.0442 | NUDT21 | 0.6807 | 0.0238 | NONO | 0.6529 | 0.0180 |
| RPL14 | 0.7056 | 0.0077 | FARSB | 0.6803 | 0.0078 | ELAVL1 | 0.6512 | 0.0223 |
| LZTS1 | 0.7055 | 0.0177 | PODXL | 0.6794 | 0.0098 | PRPF40A | 0.6511 | 0.0317 |
| LAMB2 | 0.7054 | 0.0050 | FLT1 | 0.6790 | 0.0332 | WBP11 | 0.6511 | 0.0429 |
| EFTUD2 | 0.7053 | 0.0331 | DHX15 | 0.6783 | 0.0336 | COL4A2 | 0.6509 | 0.0098 |
| RRP9 | 0.7047 | 0.0293 | HNRNPU | 0.6782 | 0.0414 | HNRNPLL | 0.6502 | 0.0095 |
| RPS13 | 0.7043 | 0.0170 | GRIA3 | 0.6776 | 0.0040 | SRSF6 | 0.6487 | 0.0118 |
| SYNCRIP | 0.7038 | 0.0195 | PLCB3 | 0.6772 | 0.0338 | VANGL2 | 0.6476 | 0.0284 |
| CPSF7 | 0.7031 | 0.0302 | HDGFL2 | 0.6756 | 0.0433 | HIST2H2AB | 0.6473 | 0.0203 |
| RPL6 | 0.7027 | 0.0205 | ILF2 | 0.6755 | 0.0374 | HNRNPL | 0.6469 | 0.0143 |
| HNRNPUL2 | 0.7019 | 0.0115 | MYH10 | 0.6737 | 0.0003 | SSB | 0.6456 | 0.0282 |
| SF3B1 | 0.7016 | 0.0286 | EWSR1 | 0.7274 | 0.0423 | TCAF2 | 0.6454 | 0.0057 |
| SAE1 | 0.7481 | 0.0408 | SAFB2 | 0.7267 | 0.0455 | GUCY1A2 | 0.6453 | 0.0126 |
| RRM1 | 0.6447 | 0.0163 | LTBP4 | 0.6092 | 0.0206 | KRR1 | 0.5527 | 0.0294 |
| RBM39 | 0.6414 | 0.0257 | HNRNPA3 | 0.6075 | 0.0105 | HIST1H1C | 0.5472 | 0.0238 |
| HNRNPM | 0.6405 | 0.0187 | XRCC6 | 0.6060 | 0.0326 | TRMT5 | 0.5391 | 0.0301 |
| NOP56 | 0.6388 | 0.0349 | DCLK2 | 0.6043 | 0.0215 | THSD7A | 0.5386 | 0.0413 |
| SRSF9 | 0.6372 | 0.0260 | HNRNPR | 0.6027 | 0.0147 | TOP2B | 0.5349 | 0.0026 |
| ATRX | 0.6367 | 0.0069 | RBM10 | 0.6022 | 0.0027 | DEFA3 | 0.5325 | 0.0361 |
| COL4A1 | 0.6353 | 0.0079 | SUGP2 | 0.6010 | 0.0017 | CBX5 | 0.5320 | 0.0028 |
| SMCHD1 | 0.6346 | 0.0173 | TOP1 | 0.6009 | 0.0104 | LRP1B | 0.5304 | 0.0042 |
| DCTPP1 | 0.6346 | 0.0181 | HNRNPA1 | 0.6008 | 0.0084 | PHF6 | 0.5249 | 0.0329 |
| DDX5 | 0.6337 | 0.0012 | RPL35 | 0.6003 | 0.0008 | HOMER1 | 0.5162 | 0.0405 |
| RPF2 | 0.6303 | 0.0182 | LAMA4 | 0.5969 | 0.0121 | HIST1H1B | 0.5138 | 0.0038 |
| XRCC5 | 0.6284 | 0.0301 | LIG3 | 0.5961 | 0.0116 | MYBBP1A | 0.5131 | 0.0121 |
| SNU13 | 0.6255 | 0.0362 | ENPEP | 0.5926 | 0.0003 | KIF21B | 0.5106 | 0.0153 |
| PHF5A | 0.6241 | 0.0248 | SUPT16H | 0.5912 | 0.0401 | GAR1 | 0.5063 | 0.0312 |
| RFC4 | 0.6224 | 0.0171 | LONP2 | 0.5890 | 0.0057 | NOVA1 | 0.4876 | 0.0038 |
| CTNNBL1 | 0.6216 | 0.0134 | SSRP1 | 0.5867 | 0.0215 | TMEM97 | 0.4852 | 0.0041 |
| PSIP1 | 0.6214 | 0.0067 | TNR | 0.5851 | 0.0045 | CHTOP | 0.4758 | 0.0221 |
| FEN1 | 0.6173 | 0.0236 | MRPS35 | 0.5795 | 0.0062 | MMP8 | 0.4492 | 0.0012 |
| LAMC1 | 0.6170 | 0.0119 | IRS2 | 0.5786 | 0.0336 | DSCAML1 | 0.4341 | 0.0006 |
| SARM1 | 0.6152 | 0.0107 | CTSG | 0.5747 | 0.0227 | S100A12 | 0.4221 | 0.0393 |
| RSL1D1 | 0.6149 | 0.0361 | ADGRL3 | 0.5742 | 0.0204 | AZU1 | 0.4207 | 0.0112 |
| RBMX | 0.6149 | 0.0163 | HNRNPA0 | 0.5741 | 0.0112 | CXCR1 | 0.4196 | 0.0006 |
| BCAN | 0.6140 | 0.0489 | U2AF1L5 | 0.5699 | 0.0104 | FBLN5 | 0.3375 | 0.0140 |
| FUS | 0.6112 | 0.0447 | DDX17 | 0.5692 | 0.0223 | S100P | 0.2661 | 0.0416 |
| HNRNPAB | 0.6107 | 0.0489 | MYEF2 | 0.5654 | 0.0071 |  |  |  |

**Supplementary Figure S1. Expression profiles of ITPKB in glioma.**


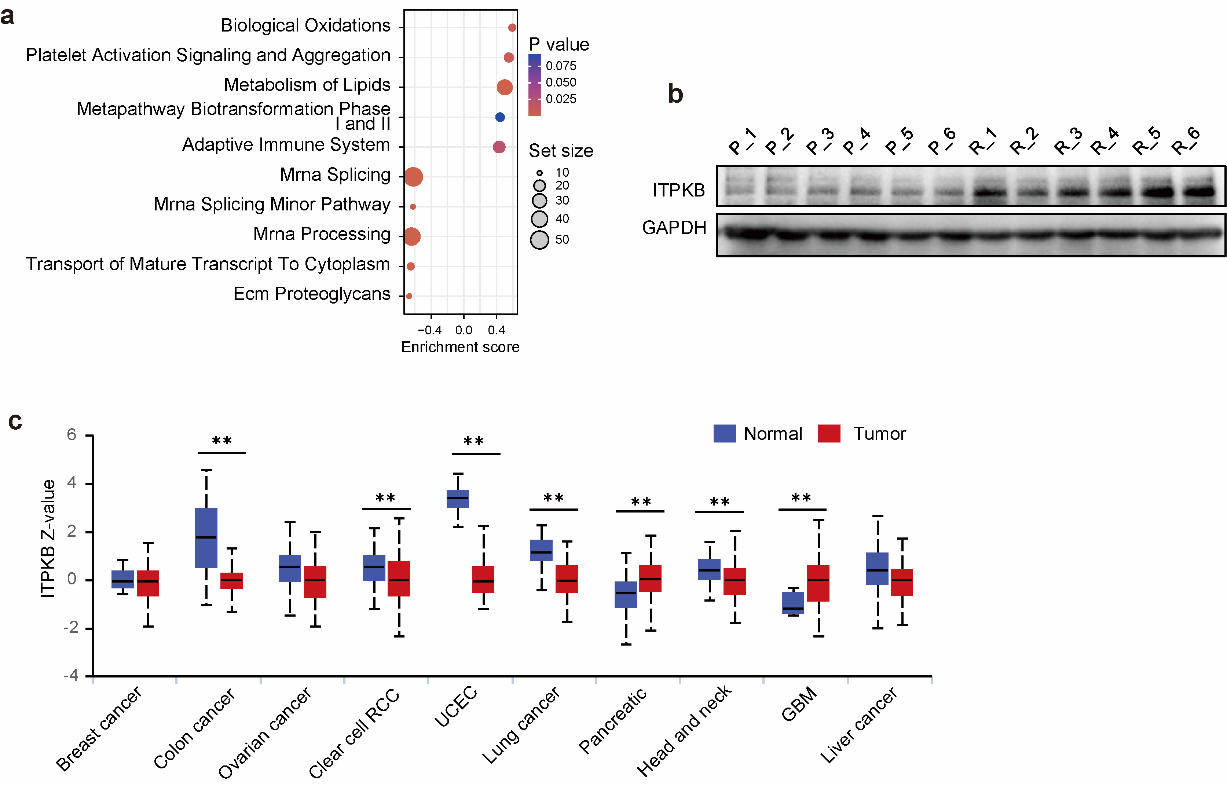


(**a**) GSEA enrichment analysis of differentially expressed proteins between primary and recurrent GBM patients. (**b**) The expression of ITPKB protein, as depicted in Figure 1E, was analyzed and validated by Western Blot. (**c**) Pan-cancer expression patterns of ITPKB (normal vs. tumor) were evaluated using the UALCAN database.

**Supplementary Figure S2. ITPKB mediates TMZ sensitivity in GBM cells through its kinase activity.**


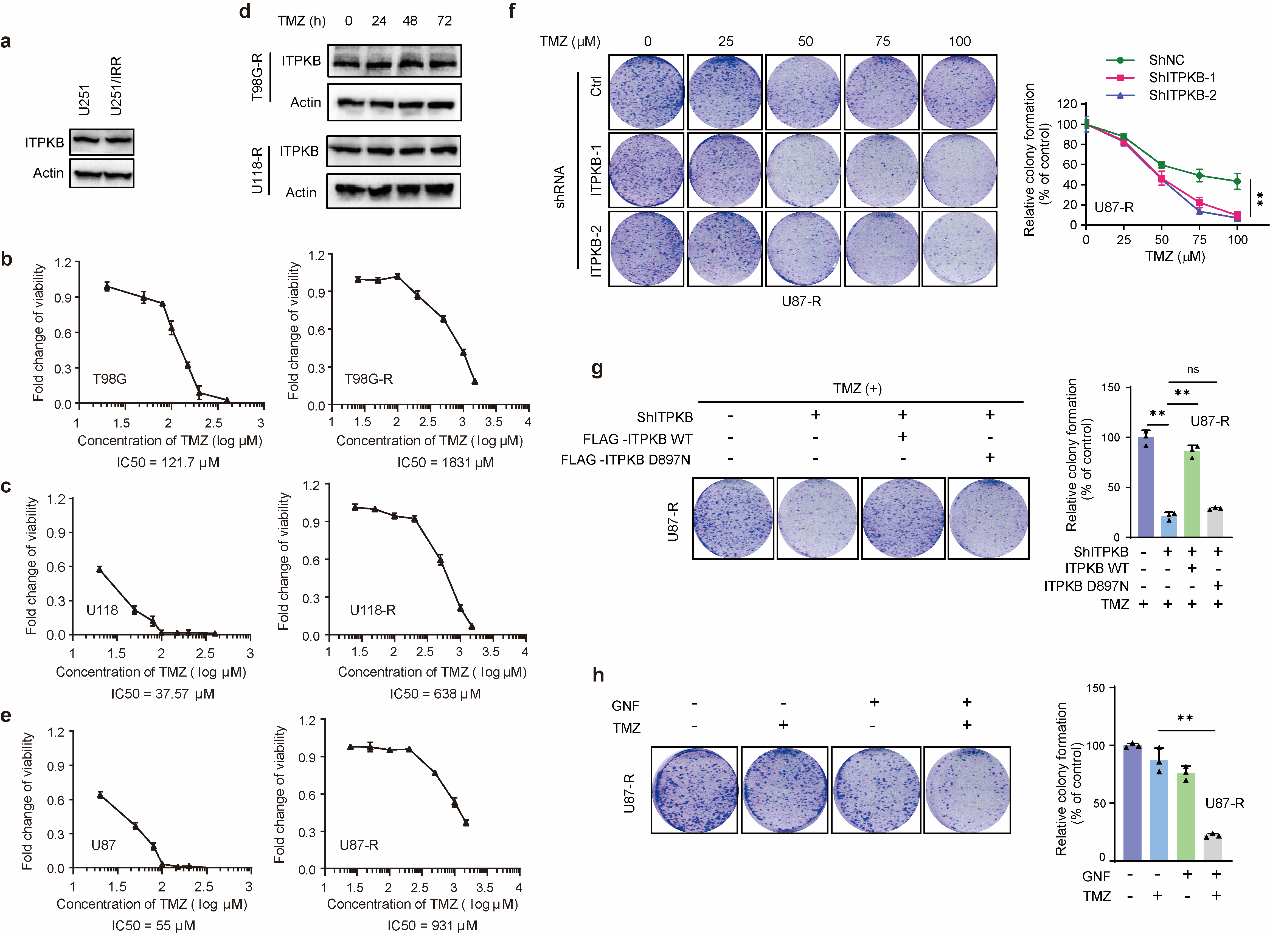


(**a**) Protein expression of ITPKB was assessed in a pair of radiation-sensitive and resistant GBM cells (U251 and U251-IR cells) using Western Blot. (**b-c**) The IC_50_ of TMZ was analyzed by CCK8 assay in T98G-R/T98G and U118-R/U118 cells. (**d**) T98G-R and U118-R cells were treated with TMZ for the indicated times, and cell lysates were blotted with specified antibodies. (**e**) The IC_50_ of TMZ was analyzed by CCK8 assay in U87-R/U87 cells. (**f**) U87-R cells were transduced with lentivirus encoding control (Ctrl) or ITPKB shRNAs and treated with 100 μM TMZ for 10-15 days. Cell survival was determined by colony formation assay. Error bars represent ± SD from three independent experiments. (**g**) U87-R cells stably expressing Ctrl or ITPKB shRNA-1 were transiently transfected with the wild-type (WT) and kinase-dead mutant D897N of ITPKB and treated with 100 μM TMZ for 10-15 days. Cell survival was determined by colony formation assay. Error bars represent ± SD from three independent experiments. (**h**) U87-R cells were treated with 20 μM GNF362 and/or 25 μM TMZ, and cell survival was determined by colony formation assay. Error bars represent ± SD from three independent experiments. Statistical significance is shown as: **p* < 0.01, ***p*<0.001, *ns*, not significant.

**Supplementary Figure S3. ITPKB involvement in TMZ sensitivity through ROS homeostasis.**


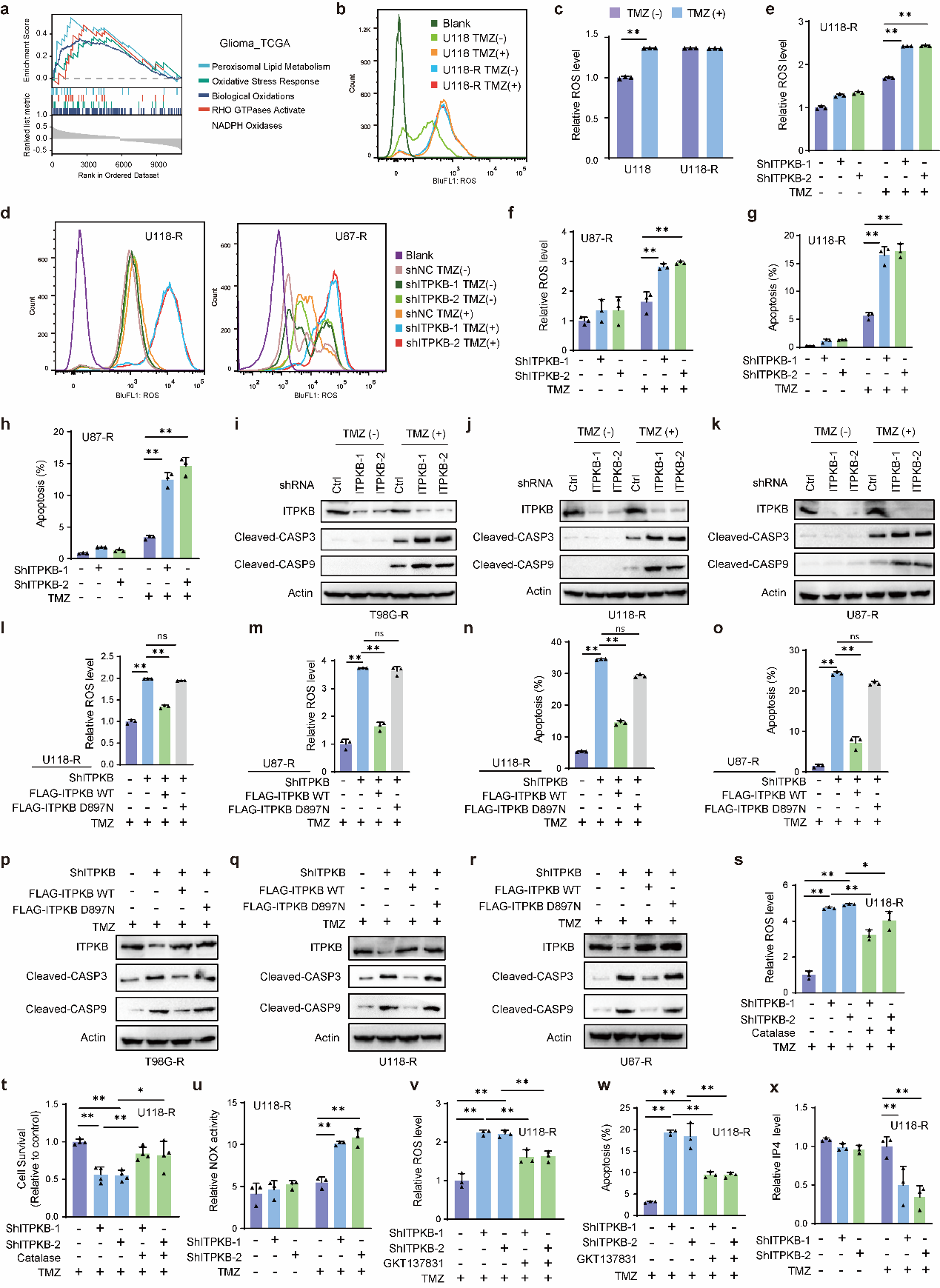


(**a**) The Linkedomics database (http://www.linkedomics.org/login.php) was utilized to analyze the glioma dataset from TCGA, focusing on the expression of ITPKB. (**b-c**) TMZ sensitive and resistant cells were incubated with 100 μM TMZ for 48 h. ROS was measured using the DCFDA assay. Data are from three independent samples and shown as mean fold change to control ± SD. (**d-h**) U118-R and U87-R cells stably expressing Ctrl or ITPKB shRNAs were treated with/without 100 μM TMZ for 48 h. ROS was measured using the DCFDA assay. After 500 μM TMZ treatment, cell apoptosis was assessed by the Annexin V-FITC apoptosis kit. Data are from three independent samples and shown as mean fold change to control ± SD. (**i-k**) TMZ-resistant glioma cells from Fig. 3e and Supplementary Fig. 3g-h were blotted with indicated antibodies. (**l-o**) TMZ resistant glioma cells stably expressing Ctrl or ITPKB shRNA-1 were transiently transfected with the wild-type (WT) and kinase-dead mutant D897N of ITPKB. After 100 μM TMZ treatment, ROS levels were determined by DCFDA assay. After 500 μM TMZ treatment, cell apoptosis was assessed by the Annexin V-FITC apoptosis kit. (**p-r**) TMZ resistant glioma cells from Fig. 3g and Supplementary Fig. 3n-o were blotted with indicated antibodies. (**s-t**) U118-R cells stably expressing Ctrl or ITPKB shRNAs were treated with 100 μM TMZ and/or 1000 U/mL antioxidant enzyme Catalase. ROS levels were measured using the DCFDA assay. Relative cell survival was determined by CCK8 assay. Error bars represent ± SD from three independent experiments. (**u**) U118-R cells stably expressing Ctrl or ITPKB shRNAs were treated with/without 100 μM TMZ for 48 h. NOX activity was analyzed by NADH Oxidase Activity Assay Kit. (**v-w**) U118-R cells stably expressing Ctrl or ITPKB shRNAs were treated with 100 μM TMZ and/or 10 μM NOX1/4 inhibitor GKT137831. ROS levels were measured using the DCFDA assay. For cell apoptosis, U118-R cells stably expressing Ctrl or ITPKB shRNAs were treated 500 μM TMZ and/or 10 μM NOX1/4 inhibitor GKT137831, and then assessed by the Annexin V-FITC apoptosis kit. (**x**) The level of IP4 was measured in U118-R cells that were stably expressing control or ITPKB shRNAs using an ELISA assay. Statistical significance is shown as: **p* < 0.01, ***p*<0.001, *ns*, not significant.

**Supplementary Figure S4. Trim25 is an ITPKB binding protein.**


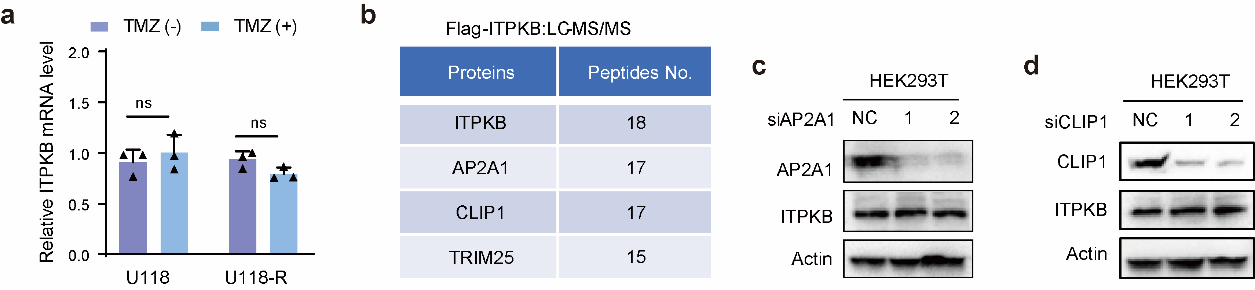


(**a**) Transcriptional mRNA levels of ITPKB were analyzed after TMZ treatment using RT-PCR. (**b**) A list of proteins binding to ITPKB was identified through mass spectrometric analysis. HEK293T cells expressing Flag-ITPKB were generated, and ITPKB complexes were subjected to mass spectrometric analysis. (**c-d**) HEK293 cells were transfected with AP2A1 and CLIP1 siRNA, and the impact on ITPKB expression was assessed by Western Blot.

**Supplementary Figure S5. ITPKB interaction with Trim25 depends on its K48 ubiquitination at sites K793 and K818.**


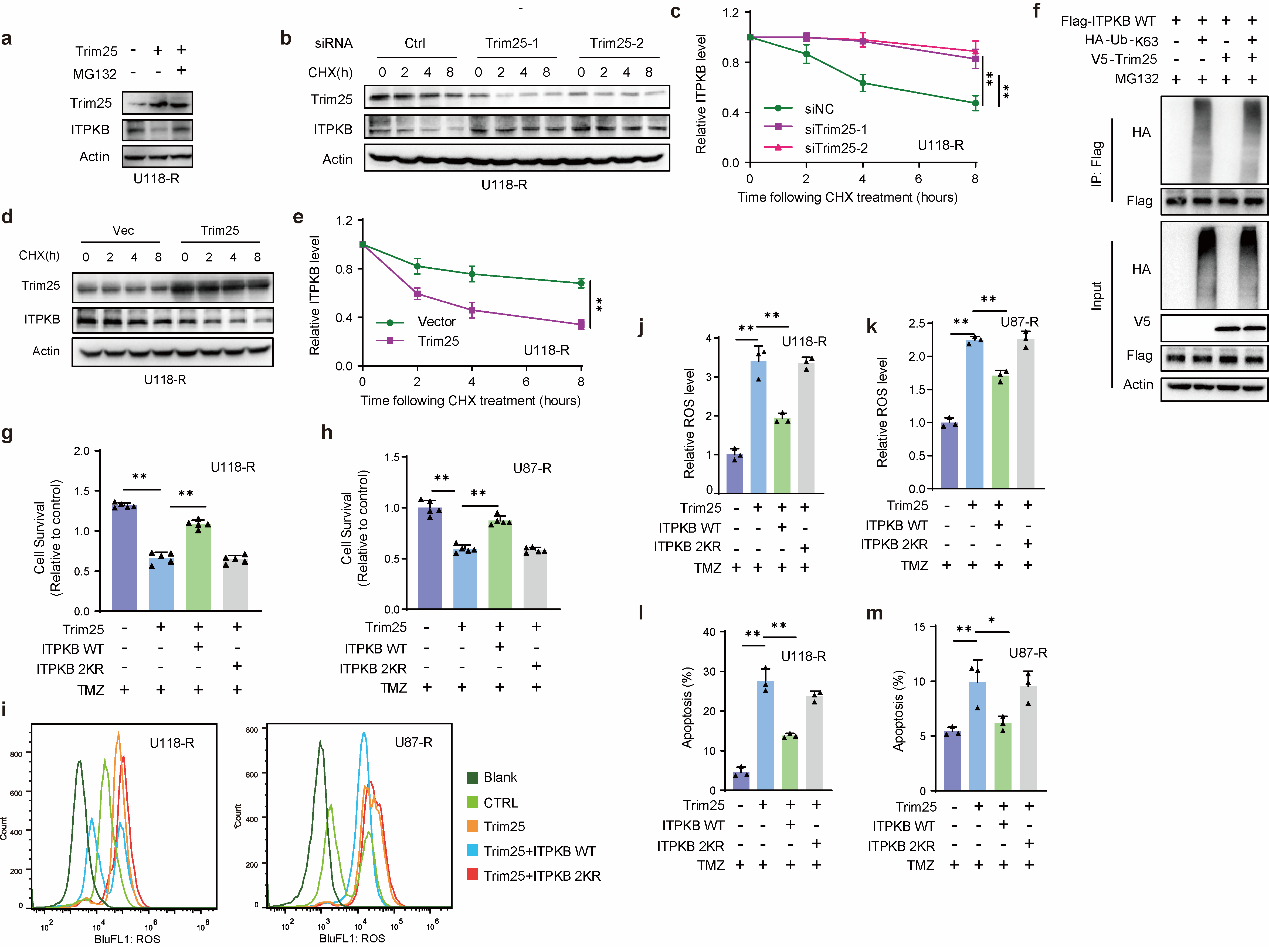


(**a**) U118-R cells transfected with Ctrl or Trim25 plasmid were treated with vehicle or MG132 (50 μM) for 3 h. Cell lysates were then blotted with indicated antibodies. (b) U118-R cells transfected with control (Ctrl) or Trim25 siRNAs were treated with cycloheximide (CHX, 0.1 mg/mL) and harvested at the indicated times. Cells were lysed, and cell lysates were then blotted with the indicated antibodies. (c) Quantification of the ITPKB protein levels relative to Actin. (d) U118-R cells transfected with Trim25 plasmid were treated with CHX (0.1 mg/mL) and harvested at the indicated times. (e) Quantification of the ITPKB protein levels relative to Actin. (f) K63 ubiquitination of ITPKB. HEK293T cells expressing Flag-ITPKB were transiently transfected with V5 tagged Trim25 and HA-tagged K63 ubiquitin. After 48 h, cells were treated with MG132 (50 μM) for 3 h. Cell lysates were subjected to immunoprecipitation with Anti-FLAG^®^ M2 Magnetic Beads, and then blotted with the indicated antibodies. (**g-m**) Trim25 plasmids were co-transfected with the control vector, ITPKB wildtype, or the 2KR mutant in U118-R and U87-R cells. ROS levels were measured using the DCFDA assay. Cell apoptosis was assessed by the Annexin V-FITC apoptosis kit. Relative cell survival was determined by CCK8 assay. Statistical significance is shown as: **p* < 0.01, ***p* < 0.001.

**Supplementary Figure S6. Trim25 protein levels in primary and recurrent GBM patients.**


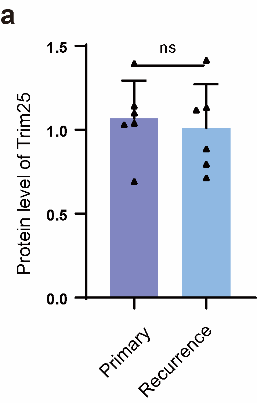

Supplement: Supplementary file 1 — Supplementary information [file 41392_2024_1763_MOESM1_ESM.docx]
